# Re-establishment of the genus *Pseudalbizzia* (Leguminosae, Caesalpinioideae, mimosoid clade): the New World species formerly placed in *Albizia*

**DOI:** 10.3897/phytokeys.205.76821

**Published:** 2022-08-22

**Authors:** Gabriela Aviles Peraza, Erik J. M. Koenen, Ricarda Riina, Colin E. Hughes, Jens J. Ringelberg, German Carnevali Fernández-Concha, Ivón Mercedes Ramírez Morillo, Lilia Lorena Can Itza, Ivan Tamayo-Cen, Jorge Humberto Ramírez Prado, Xavier Cornejo, Sawai Mattapha, Rodrigo Duno de Stefano

**Affiliations:** 1 Herbarium CICY, Centro de Investigación Científica de Yucatán, A.C. (CICY), Calle 43 No. 130, Col. Chuburna de Hidalgo, 97200, Mérida, Yucatán, Mexico Centro de Investigación Científica de Yucatán Mérida Mexico; 2 Evolutionary Biology & Ecology, Université Libre de Bruxelles, Av. F.D. Roosevelt, 50, CP 160/12, Brussels B-1050, Belgium Université Libre de Bruxelles Brussels Belgium; 3 Real Jardín Botánico, CSIC. Plaza de Murillo, 2. Madrid 28014, Spain Real Jardín Botánico, RJB-CSIC Madrid Spain; 4 Department of Systematic and Evolutionary Botany, University of Zurich, Zollikerstrasse 107, Zurich CH-8008, Switzerland University of Zurich Zürich Switzerland; 5 Orchid Herbarium of Oakes Ames, Harvard University Herbaria, 22 Divinity Avenue, Cambridge, Massachusetts 02138, USA University of Zurich Zurich Switzerland; 6 Unidad Biotecnología Centro de Investigación Científica de Yucatán, A.C. (CICY), Calle 43 No. 130, Col. Chuburna de Hidalgo, 97200, Mérida, Yucatán, Mexico Harvard University Herbaria Cambridge United States of America; 7 Herbario GUAY, Facultad de Ciencias Naturales, Universidad de Guayaquil, Avenida Juan Tanca Marengo s/n y Avenida de las Aguas Casilla 09-01-10634, Guayaquil, Ecuador Universidad de Guayaquil Guyaquil Ecuador; 8 Department of Biology, Faculty of Science, Udon Thani Rajabhat University, Udon, 41000 Thailand Udon Thani Rajabhat University Udon Thailand

**Keywords:** *Arthrosamanea*, hydrochory, monophyly, Neotropics, phylogeny, taxonomy

## Abstract

Following recent mimosoid phylogenetic and phylogenomic studies demonstrating the non-monophyly of the genus *Albizia*, we present a new molecular phylogeny focused on the neotropical species in the genus, with much denser taxon sampling than previous studies. Our aims were to test the monophyly of the neotropical section Arthrosamanea, resolve species relationships, and gain insights into the evolution of fruit morphology. We perform a Bayesian phylogenetic analysis of sequences of nuclear internal and external transcribed spacer regions and trace the evolution of fruit dehiscence and lomentiform pods. Our results find further support for the non-monophyly of the genus *Albizia*, and confirm the previously proposed segregation of *Hesperalbizia*, *Hydrochorea*, *Balizia* and *Pseudosamanea*. All species that were sampled from section Arthrosamanea form a clade that is sister to a clade composed of *Jupunba*, *Punjuba*, *Balizia* and *Hydrochorea*. We find that lomentiform fruits are independently derived from indehiscent septate fruits in both *Hydrochorea* and section Arthrosamanea. Our results show that morphological adaptations to hydrochory, associated with shifts into seasonally flooded habitats, have occurred several times independently in different geographic areas and different lineages within the ingoid clade. This suggests that environmental conditions have likely played a key role in the evolution of fruit types in *Albizia* and related genera. We resurrect the name *Pseudalbizzia* to accommodate the species of section Arthrosamanea, except for two species that were not sampled here but have been shown in other studies to be more closely related to other ingoid genera and we restrict the name *Albizia* s.s. to the species from Africa, Madagascar, Asia, Australia, and the Pacific. Twenty-one new nomenclatural combinations in *Pseudalbizzia* are proposed, including 16 species and 5 infraspecific varietal names. In addition to the type species *Pseudalbizziaberteroana*, the genus has 17 species distributed across tropical regions of the Americas, including the Caribbean. Finally, a new infrageneric classification into five sections is proposed and a distribution map of the species of *Pseudalbizzia* is presented.

## Introduction

**Figure 1.**
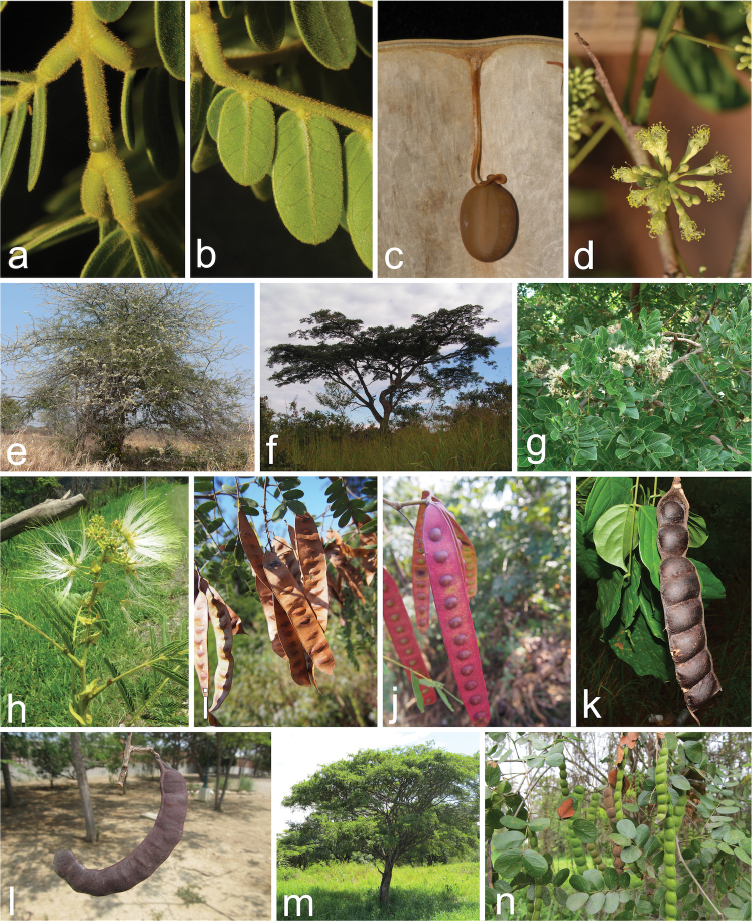
Morphology of *Albizia* s.l. showing selected members of the genera *Albizia* and *Pseudalbizzia***a–c***Albiziaferruginea* (Guill. & Perr.) Benth. in Congo **a** detail of leaf rachis and gland between terminal pinnae **b** detail of leaflets of a terminal pinna **c** seed and funiculus attached to the valve **d***Albiziaglaberrima* (Schumach. & Thonn.) Benth. in Malawi, detail of inflorescence **e***Albiziaanthelmintica* Brongn. in Malawi, habit **f***Albiziaadianthifolia* (Schumach.) W. Wight in Congo, habit **g***Albiziaglaberrima* in Malawi, branches and inflorescences **h***Albiziachinensis* (Osbeck) Merr. in Thailand, inflorescences **i***Albiziaodoratissima* (L. f.) Benth. in Thailand, fruits **j***Albiziaprocera* (Roxb.) Benth. in Thailand, fruits **k***Albiziasplendens* Miq. in Thailand, woody fruit **l**Pseudalbizziamultifloravar.multiflora in Ecuador, woody fruit **m, n***Pseudalbizziapistaciifolia* (Willd.) E.J.M Koenen & Duno in Ecuador **m** habit **n** woody fruit. Photos: **a, b** David J. Harris / With permission from RBG Edinburgh **c** Claude Boucher Chisale **d–f** Günter Baumann **g** Jos Stevens **h** Natcha Sutjaritjai **i–k** Prateep Panyadee **l–n** Xavier Cornejo.

**Figure 2.**
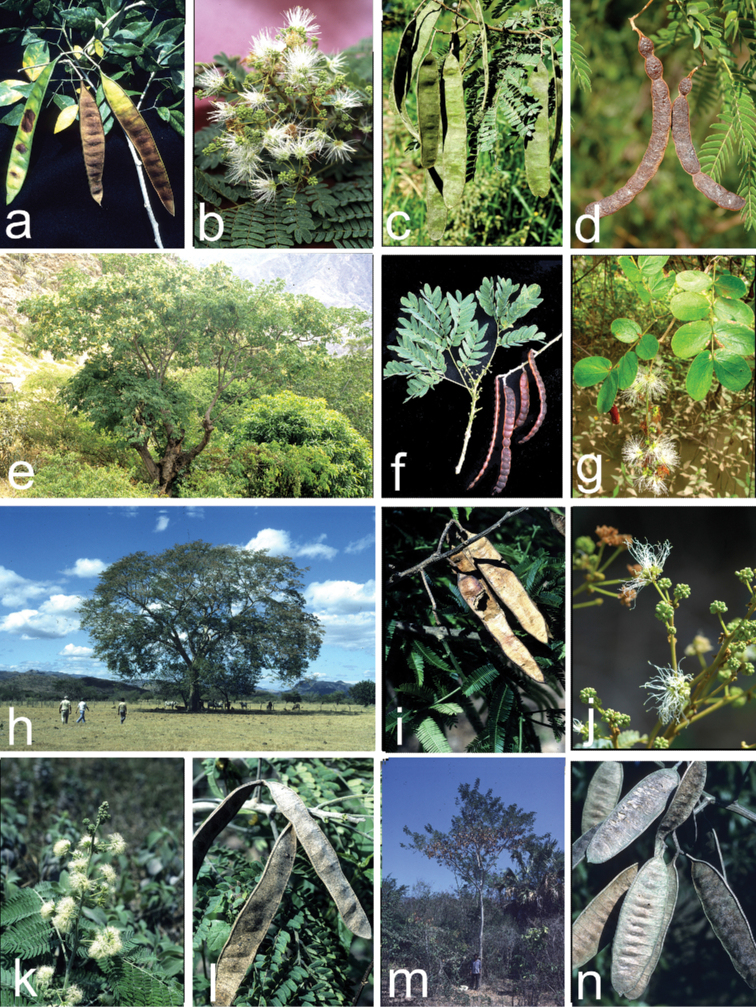
Habit, flower and fruit variation in the genus *Pseudalbizzia***a***P.adinocephala* pods (*Hughes 1913*) **b***P.coripatensis* inflorescence (*Hughes 2433*) **c***P.coripatensis* pods (*Hughes 2433*) **d***P.inundata* pods (*JRI Wood 26530*) **e***P.multiflora* habit (*Hughes 2214*) **f***P.multiflora* leaves and pods (*Hughes 2214*) **g***P.pistaciifolia* leaves and inflorescence (*Cornejo 8426, GUAY*) **h***P.niopoides* habit (*Hughes 419*) **i***P.niopoides* pods (*Rivera 2245*) **j***P.polycephala* inflorescence (*de Queiroz 15515*) **k***P.tomentosa* inflorescence (*Hughes 1143*) **l***P.sinaloensis* pods (*Hughes 1576*) **m***P.tomentosa* habit (*Hughes 1335*) **n***P.tomentosa* pods (*Hughes 1307*). All photos by Colin Hughes except **g**, Xavier Cornejo.

The genus *Albizia* Durazz. has a complicated taxonomic history but has generally been treated as a pantropical genus with 120–140 species, of which 36 are endemic to Africa, with c. 30 species in Madagascar, of which c. 24 are endemic, c. 35 species in Asia, one in Australia, and 22 in tropical America (Lewis and Rico Arce 2005; Rico Arce et al. 2008). All species are woody, forming trees of variable stature and inhabit a wide range of lowland tropical biomes (Figs 1 and 2), including rain forests, seasonally dry tropical forests, and savannas, with one species, *Albiziajulibrissin* Durazz., the type species of the genus, in subtropical and warm temperate forests in Asia. However, *Albizia* remains poorly defined; its delimitation remains one of the most challenging taxonomic problems in the legume family, and it is currently considered the main “dustbin” genus in tribe Ingeae (Koenen et al. 2020). In the past, the most problematic genus of tribe Ingeae was *Pithecellobium* Mart., but its taxonomy has been gradually clarified (Barneby and Grimes 1996). Resolution of the taxonomic status of *Albizia* has lagged behind that of *Pithecellobium* and only really started at the end of the twentieth century. For example, several new neotropical genera have been segregated from *Albizia*: *Balizia* Barneby & J.W. Grimes; *Hesperalbizia* Barneby & J.W. Grimes, and *Hydrochorea* Barneby & J.W. Grimes. Barneby and Grimes (1996) also re-established the genus *Pseudosamanea* Harms, which previously had been treated as a synonym within *Albizia* (Table 1). However, at the time they were established, the monophyly of these new and re-established genera had not been tested using phylogenetic analyses of molecular data.

**Table 1.** Main taxonomic changes related to *Albizia*, Ingeae tribe [1981–2008]. Modified from Rico Arce et al. (2008).

Nielsen (1981)	Barneby and Grimes (1996)	Lewis and Rico Arce (2005)	Rico Arce et al. (2008)	Iganci et al. 2015
***Albizia***	***Albizia***	***Albizia***	***Albizia***	***Albizia***
	***Balizia***			***Balizia***
***Cathormion***	***Cathormion***	***Cathormion***	*	*
	***Hydrochorea***		*	***Hydrochorea***
	***Hesperalbizia***	***Hesperalbizia***		
	***Pseudosamanea***	***Pseudosamanea***		

* Not explicitly mentioned in the study.

Barneby and Grimes (1996) placed the remaining New World species of Albizia in their section Arthrosamanea (Britton & Rose) Barneby & J.W. Grimes. They characterized this section as forming a group that is homogeneous in most respects, but diverse in the late developmental stages of the fruit, which vary in: 1) fruit opening type: dehiscent, indehiscent, or breaking, 2) lateral shape: flat to conspicuously raised above the seed chambers, 3) texture and consistency of the valves: papery, chartaceous or woody, 4) longitudinal shape: straight to weakly falcate (Barneby and Grimes 1996) (Figs 1, 2 and 4). Within section Arthrosamanea, four series were proposed by Barneby and Grimes (1996): series *Paniculatae* with papery, plano-compressed, inertly dehiscent pods with continuous valves (13 species); series *Arthrosamanea* comprising 3 species with lomentiform, plano-compressed pods, where the ripe valves crack transversely between seeds but the wiry sutures persist at maturity; series *Multiflorae* (2 species) characterized by lomentiform fruits only reluctantly separating into articles, the thick-textured valves and sutural keels breaking transversely under pressure; and the monospecific series *Inundatae* which bears crypto-lomentiform pods, dehiscent through the sutures and with the valves differentiating into a continuous exocarp and a segmented endocarp separating into 1-seeded segments (Barneby and Grimes 1996).

Series *Paniculatae* is widespread across Mexico, Central and South America, occurring mainly in seasonally dry forests, grasslands, and less often in humid forests (in South America). All species of series *Paniculatae* have papyraceous, dehiscent fruits with one exception, *A.berteroana* (DC.) Fawc. & Rendle (the earlier combination *A.berteroana* (DC.) M. Gómez was invalidly published due to incorrect citation of the basionym, see Barneby and Grimes 1996), whose fruits are indehiscent and fall to the ground entire. In contrast, the other three series are distributed from Panama to South America and are most diverse in the Amazon basin (Barneby and Grimes 1996), and usually have more or less woody fruits, which are articulated and indehiscent, some dividing into monospermous segments through the grooves of the valves, considered to be an adaptation for hydrochory, i.e., seed dispersal in riparian and seasonally inundated forests (*e.g.*, *A.inundata* (Mart.) Barneby & J.W. Grimes, *A.pistaciifolia* (Willd.) Barneby & J.W. Grimes, and *A.subdimidiata* (Splitg.) Barneby & J.W. Grimes).

The segregate genera established by Barneby and Grimes (1996) have not been universally accepted. For example, in the most recent taxonomic treatment of *Albizia* for Mexico and Central America (Rico Arce et al. 2008), the genera *Balizia*, *Hesperalbizia*, and *Pseudosamanea* were not recognized (Table 1). However, subsequent phylogenetic analyses have confirmed that these genera were rightfully segregated as distinct evolutionary lineages, *Hesperalbizia* being more closely related to *Lysiloma* Benth. (Duno de Stefano et al. 2021), and the closely related *Balizia* and *Hydrochorea* (the former reduced to synonymy of the latter, Soares et al. 2022) being placed as the sister-group of *Jupunba* Britton & Rose (Iganci et al. 2015; Soares et al. 2021, 2022). Phylogenomic analysis of the mimosoid clade, based on DNA sequences of 964 targeted nuclear genes confirmed these findings and, furthermore, showed that the species from the Americas (i.e., sect. Arthrosamanea) form a separate lineage from the African, Madagascan and Asian species (Koenen et al. 2020). Koenen et al. (2020) also showed that *Pseudosamanea*, although difficult to place in any clade, is not closely related to either Albizia s.s. or section Arthrosamanea and is perhaps most closely related to *Samanea* Merr. and *Chloroleucon* Britton & Rose ex Record.

While the data of Koenen et al. (2020) provided a robust phylogenomic backbone for the mimosoid and ingoid clades, and clearly demonstrated the non-monophyly of *Albizia* by sampling 25 species of that genus, only three of the 19 species from Central and South American sect. Arthrosamanea were included in that study, leaving doubts about the monophyly of that section and whether it should be segregated under the circumscription of Barneby and Grimes (1996), or whether there are further potential segregates, given the possibility that some of these species are more closely related to other neotropical genera. That possibility was suggested by the occurrence of lomentiform fruits in some species of section Arthrosamanea as reflected in the classification into separate series by Barneby and Grimes (1996). Similar lomentiform fruits also occur in *Hydrochorea* (Barneby and Grimes 1996; Soares et al. 2022) and *Albizia* s.s. (*Albiziadolichadena* (Kosterm.) I.C. Nielsen, *Albiziamoniliformis* (DC.) F. Muell., *Albiziarosulata* (Kosterm.) I.C. Nielsen and *Albiziaumbellata* (Vahl) E.J.M. Koenen), as well as a few other ingoid lineages (Barneby and Grimes 1996: 204; Koenen 2022a). For this reason, several species of neotropical *Albizia* have homotypic synonyms in *Arthrosamanea* Britton & Rose, *Samanea* or *Cathormion* Hassk., genera which previously had been recognized and defined based mainly on characters of fruit texture and dehiscence (Barneby and Grimes 1996). Barneby and Grimes (1996: 204) considered these fruit types to have arisen multiple times in parallel in different genera, and this was confirmed by subsequent phylogenetic (Iganci et al. 2015; Soares et al. 2021) and phylogenomic studies (Koenen et al. 2020), although the neotropical lomentiform *Albizia* species were not included in these studies, or remained unresolved.

Here we investigate whether Albiziasect.Arthrosamanea is monophyletic and thereby provide a more rigorous basis for recognizing its evolutionary distinctiveness from *Albizia* s.s. as a segregate genus. We infer a new phylogeny with emphasis on the neotropical species and make use of further insights offered by the phylogenomic analysis of Ringelberg et al. (2022). In addition, we use a tree topology inferred from data from the latter study to evaluate whether lomentiform fruits in Albiziasect.Arthrosamanea are independently derived from other lineages in which this fruit type occurs. Based on our phylogenetic results and the recent findings of Koenen et al. (2020) and Ringelberg et al. (2022), we update the taxonomy of neotropical *Albizia* by resurrecting the genus *Pseudalbizzia* Britton & Rose.

## Materials and methods

We used the nuclear ribosomal External and Internal Transcribed Spacer (ETS and ITS) regions that previously been used to study sister-group relationships within tribe Ingeae (Brown et al. 2008; Iganci et al. 2015; Souza et al. 2016). Our combined dataset included 123 accessions, of which 50 are from Genbank and 73 are newly sequenced here, including 25 species of *Albizia* s.l. sequenced for the first time. The outgroup, *Vachelliafarnesiana* (L.) Wight & Arn., was designated to root the tree (Table 2). The plastid *trnK* region was initially explored but preliminary analyses suggested it is not sufficiently phylogenetically informative and these data were excluded from this study.

Fresh leaf material collected in the field plus herbarium material from the Jardín Botánico Regional Roger Orellana (CICY) were used for DNA extraction. Herbarium specimens used in these analyses came from AAU, CICY, FCME, MA, MEXU, and MO (acronyms as in Thiers 2016). Additional sequences were downloaded from GenBank (Table 2).

**Table 2.** Voucher information of taxa included in the phylogenetic analysis with their corresponding GenBank accession numbers.

Accessions ITS	Accessions ETS
*Albiziaadianthifolia* (Schumach.) W. Wight, MW699934, BGRO 001	*Albiziaadianthifolia*, MW699372, BGRO 001
*Albiziaamara* (Roxb.) Boivin, MW699936, BGRO 003	*Albiziaamara*, MW699374, BGRO 003
*Albiziaanthelmintica* Brongn., MW699937, BGRO 004	*Albiziaanthelmintica*, MW699375, BGRO 004
*Albiziaarenicola* R. Vig., MW699938, R. Randrianaivo 642, MO	*Albiziaantunesiana* Harms, MW699376, S.H.C.P. 966, MO
*Albiziabrevifolia* Schinz, MW699940, BGRO 005	*Albiziaarenicola*, MW699377, R. Randrianaivo 642, MO
*Albiziaglaberrima* Hutch. & Dalziel, MW699943, R.E. Gereau 6203, MA	*Albiziabrevifolia*, MW699378, BGRO 005
*Albiziagummifera* (J.F. Gmel.) C.A. Sm., MW699944, J.E. Lawesson 5094, AAU	*Albiziachinensis* (Osbeck) Merr., MW69379, A. Ntemi & A. Athumani 478, MO
*Albiziaharveyi* E. Fourn., MW699945, BGRO 006	*Albiziacrassiramea* Lace, MW699380, K. Larsen et al. 46378, AAU
*Albiziajulibrissin* Durazz., MW699946, BGRO 007	*Albiziaferruginea* (Guill. & Perr.) Benth., MW699382, *C.H. Jongkind 2098*, MA
*Albiziakalkora* (Roxb.) Prain, MW699947, E. Bouflord 26356, MO	*Albiziaglaberrima*, MW699383, R.E. Gereau 6203, MA
*Albizialebbeck* (L.) Benth., MW699948, C. Chan 7539, CICY	*Albiziagummifera*, MW699384, J.E. Lawesson 5094, AAU
*Albiziapetersiana* (Bolle) Oliv., MW699950, BGRO 008	*Albiziaharveyi*, MW699385, BGRO 006
*Albiziaprocera* (Roxb.) Benth., MW699953, BGRO 009	*Albiziajulibrissin*, MW699387, BGRO 007
*Albiziaretusa* Benth., MW699954, K. Yasuda 1804, MO	*Albiziakalkora*, MW699388, E. Bouflord 26356, MO
*Albiziatanganyicensis* Baker f., MW699956, BGRO 010	*Albizialebbeck* (L.) Benth., MW699389, C. Chan 7539, CICY
*Albiziaumbellata* (Vahl) E. J. M. Koenen, EF638182.1	*Albizialebbekioides* (DC.) Benth., MW699390, H. Balslev 9333, AAU
*Balizialeucocalyx* (Britton & Rose) Barneby & J.W. Grimes, MW699959, S. Aguilar & F. Aguilar 1833, M	*Albizialucidior* (Steud.) I.C. Nielsen ex H. Hara, MW699391, J.F. Maxwell 95–259, MO
*Lysilomaacapulcense* (Kunth) Benth., MW699960, H. Gómez D. 2003, MO	*Albiziapetersiana* (Bolle) Oliv., MW699394, BGRO 008
*Lysilomalatisiliquum* (L.) Benth., MW699961, P. Simá 2287, CICY	*Albiziaprocera*, MW699396, BGRO 009
*Pseudalbizziaadinocephala* (Donn. Sm.) E.J.M. Koenen & Duno, MW699935, BGRO 002	*Albiziaretusa*, MW699397, K. Yasuda 1804, MO
MW699958, J.L. Linares 5406, FCME	*Albiziasahafariensis* Capuron, MW699398, R. Randrianaivo et al. 1387, MO
*Pseudalbizziaberteroana* (Balb. Ex DC.) Britton & Rose, MW699939, *A.* Jimenez 2113, MO	*Albiziatanganyicensis*, MW699400, BGRO 010
*Pseudalbizziaedwallii* (Hoehne) E.J.M. Koenen & Duno, MW699942, J.M. Silva & L.M. Abe 4237, MEXU	*Albiziaumbellata*, EF638157.1
*Pseudalbizziamultiflora* (Kunth) E.J.M. Koenen & Duno, MW699949, X. Cornejo 1922, GUAY	*Balizialeucocalyx*, MW699403, S. Aguilar & F. Aguilar 1833, M
*Pseudalbizziapistaciifolia* (Willd.) E.J.M. Koenen & Duno, MW699951, X. Cornejo 5323, GUAY	*Baliziapedicellaris* (DC.) Barneby & J.W. Grimes, MW699404, P.R. House 1880, MA
*Pseudalbizziapolycephala* (Benth.) E.J.M. Koenen & Duno, MW699952, L.P. Queiroz 9578, MEXU	*Havardiamexicana*, MW699405, S. Foldi s.n., CICY
*Pseudalbizziasinaloensis* (Britton & Rose) E.J.M. Koenen & Duno, MW699955, C.E. Hughes et al. 1576, FCME	*Hesperalbiziaoccidentalis*, MW699406, J.G. Hernandez Oria 21, FCME
*Pseudalbizziatomentosa* (Micheli) E.J.M. Koenen & Duno, MW699957, *A.* Dorantes et al. 165, CICY	*Lysilomaacapulcense*, MW699407, H. Gómez D. 2003, MO
*Pseudosamaneacubana* (Britton & P. Wilson) Barneby & J.W. Grimes, MW699941, GHBG 001	*Lysilomalatisiliquum*, MW699408, P. Simá 2287, CICY
*Pseudosamaneaguachapele* (Kunth) Harms, MW699962, BGRO 011	*Paraseriantheslophantha*, MW699409, H. Balslev et al. 62450, AAU
*Zapotecaformosa* (Kunth) H.M. Hern., MW699963, R. Duno s.n. CICY. Additional accessions (ITS): *Acaciaacradenia* F.Muell., AF487765.1	*Pithecellobiumdiversifolium*, MW699410, J.F.B. Pastore & R.M. Harley 2599 MO
*Acacialongifolia* (Andrews) Willd., HM007655.1	*Pithecellobiumexcelsum*, MW699411, G. P. Lewis et al 2339, MO
*Acaciellaangustissima* (Mill.) Britton & Rose, EF638169.*1*	*Pseudalbizziaadinocephala*, MW699373, BGRO 002
*Baliziapedicellaris* (DC.) Barneby & J.W. Grimes, JX870657.1	MW699402, J.L. Linares 5406, FCME
*Calliandradysantha* Benth., JX870684.1	*Pseudalbizziaedwallii*, MW699381, *J.M. Silva & L.M. Abe 4237*, MEXU
*Calliandrafoliosa* Benth., EF638181.1	*Pseudalbizziainundata* (Mart.) E.J.M. Koenen & Duno, MW699386, H. Balslev et al. 97355, AAU
*Cojobaarborea* (L.) Britton & Rose, JX870758.1	*Pseudalbizziamultiflora* (Kunth) E.J.M. Koenen & Duno, MW699392, X. Cornejo & T. Andres 8705, GUAY
*Cojobaundulatomarginata* L. Rico, EF638187.1	*Pseudalbizzianiopoides* (Spruce ex Benth.) E.J.M. Koenen & Duno, MW699393, J.R. Grande 374, VEN
*Ebenopsisebano* (Berland.) Barneby & J.W. Grimes, JX870759.1	*Pseudalbizziapolycephala*MW699395, L.P. Queiroz 9578, MEXU
*Enterolobiumcontortisiliquum* (Vell.) Morong, EF638190.1	*Pseudalbizziasinaloensis*, MW699399, C.E. Hughes et al. 1576, FCME
*Enterolobiumcyclocarpum* (Jacq.) Griseb., EF638191.1	*Pseudalbizziatomentosa*, MW699401, A. Dorantes et al. 165, CICY
*Enterolobiumtimbouva* Mart., JX870760.1	*Pseudosamaneacubana* (Britton & P. Wilson) Barneby & J.W. Grimes, MW699412, BJ FTGH 2000
*Faidherbiaalbida* (Delile) A. Chev., EU812008.1	*Pseudosamaneaguachapele*, MW699413, BGRO 011
*Havardiamexicana* (Rose) Britton & Rose, JX870762.1	*Samaneatubulosa* (Benth.) Barneby & J.W. Grimes, MW699414, *G.A. Parada & V.D. Rojas* 2480, MO. Additional accessions: *Acaciaacradenia*, EF638116.1
*Havardiapallens* (Benth.) Britton & Rose, KF921656.1	*Acacialongifolia*, EF638115.1
*Hesperalbiziaoccidentalis* (Brandegee) Barneby & J.W. Grimes, EF638195.*1*	*Acaciellaangustissima* EF638082.1
*Hycrochoreacorymbosa* (Rich.) Barneby & J.W. Grimes, JX870763.1	*Pseudalbizziaadinocephala* EF638144.1
*Jupunbatrapezifolia* (Vahl.) Moldenke, EF638166.1	*Albiziakalkora* EF638158.1
*Mariosousacoulteri* (Benth.) Seigler & Ebinger, EF638198.1	*Albizialebbeck* EF638155.1
*Mariosousadolichostachya* (S.F. Blake) Seigler & Ebinger, EF638199.1	*Albiziasaponaria* (Lour.) Blume, EF638085.1
*Paraseriantheslophantha* (Willd.) I.C. Nielsen, EF638204.1	*Archidendropsisbasaltica* (F. Muell.) I.C. Nielsen, EF638141.1
*Pithecellobiumdiversifolium* Benth., JX870768.1	*Archidendropsisthozetiana* (F. Muell.) I.C. Nielsen, EF638140.1
*Pithecellobiumdulce* (Roxb.) Benth., EF638207.1	*Calliandradysantha* EF638121.1
*Pithecellobiumexcelsum* (Kunth) Mart., EF638208.1	*Calliandrafoliosa* EF638122.1
*Samaneasaman* (Jacq.) Merr., JX870770.1	*Cojobaarborea* EF638095.1
*Samaneatubulosa* (Benth.) Barneby & J.W. Grimes, EF638212.1	*Cojobaundulatomarginata* EF638096.1
*Pseudosamaneaguachapele* (Kunth) Harms, JX870769.1	*Ebenopsisconfinis* (Standl.) Britton & Rose, EF638100.1
*Senegaliaberlandieri* (Benth.) Britton & Rose, KY688777.*1*	*Ebenopsisebano* EF638101.1
*Sphingaacatlensis* (Benth.) Barneby & J.W. Grimes, EF638214.1	*Enterolobiumcontortisiliquum* EF638151.1
*Vachelliacampechiana* (Mill.) Seigler & Ebinger, EF638215.1	*Enterolobiumcyclocarpum* EF638149.1
*Vachelliafarnesiana* (L.) Wight & Arn., EF638219.1	*Faidherbiaalbida* EF638163.1
*Viguieranthusambongensis* (R. Vig.) Villiers, JX870773.1	*Havardiapallens* EF638146.1
*Viguieranthusdensinervus* Villiers, JX870774.1	*Hesperalbiziaoccidentalis* EF638139.1
*Viguieranthusmegalophyllus* (R. Vig.) Villiers, JX870776.1	*Hycrochoreacorymbosa* EF638138.1
*Viguieranthussubauriculatus* Villiers, JX870778.1	*Jupunbatrapezifolia* (Vahl.) Moldenke, EF638110.1
*Zapotecatetragona* (Willd.) H.M. Hern., JX870784.1	*Mariosousacoulteri* (Benth.) Seigler & Ebinger, EF638124.1
	*Mariosousadolichostachya* EF638084.1
	*Pararchidendronpruinosum* (Benth.) I.C. Nielsen, EF638129.1
	*Paraserianthestoona* (Bailey) I.C. Nielsen, EF638106.1
	*Pithecellobiumdulce* EF638142.1
	*Pseudosamaneaguachapele* EF638160.1
	*Samaneasaman* EF638136.1
	*Samaneatubulosa* EF638135.1
	*Senegaliaberlandieri* EF638162.1
	*Sphingaacatlensis* EF638145.1
	*Vachelliafarnesiana* EF638128.1
	*Viguieranthusambongensis* KR997873.1
	*Viguieranthusdensinervus* JX870891.1
	*Viguieranthusmegalophyllus* KR997871.1
	*Viguieranthussubauriculatus* KR997076.1
	*Zapotecaformosa* EF638134.1
	*Zapotecatetragona*EF638133.1.

DNA from leaf fragments was obtained using the DNeasy Plant Mini Kit (QIAGEN Inc., Valencia, California) following the manufacturer’s specifications. To assess concentration and relative quality of DNA, 3 µl of ﬁnal volume plus 2 µl loading buffer were run for 30 minutes at 6 V cm^-1^ on a 1% agarose gel prepared with 0.5× TBE. The resulting gel was developed by immersion for 20–30 minutes in a 0.1 µg ml^-1^ ethidium bromide solution and later observed in a DigiDoc-It Imaging System (version 6.7.1; UVP, Inc., Cambridge, UK) transilluminator. DNA purity and concentration were quantified with a NanoDrop 2000c. Afterwards, DNA samples were standardized to 10 ng µl^-1^.

PCR amplifications were performed in an Applied Biosytems Veriti 96 Well Thermal Cycler. Volumes of reagents and conditions for the amplifications were as follows: **ITS**: 30 µL of mix containing 3 µl 10× Buffer, 2.5 µl MgCl_2,_, 0.6 µl (~10 ng) primer, 4 µl Q solution, 1 µl 1.25 mM L^-1^ dNTP, 0.2 µl (1 U) TAQ polymerase, 2 µl (~10 ng) DNA, then completed to volume (approx. 16.1 µl) with ultra-pure water. PCRs were conducted under the following protocol: 94 °C × 3 min + 30 cycles (94 °C × 1 min + 60.5 °C × 1 min + 72 °C × 2 min) + 72 °C × 7 min. Primers were S3 (AACCTGCGGAAGGATCATTG) (Käss and Wink 1997), and 26S (TAGAATTCCCCGGTTCGCTCGCCGTTAC) (Sun et al. 1994). **ETS**: 30 µl of mix containing 3 µl 10× Buffer, 2.5 µl MgCl_2_, 0.6 µl (~10 ng) primer, 4 µl Q solution, 1 µl 1.25 mM l^-1^ dNTP, 0.2 µl (1 U) TAQ polymerase, 2 µl (~10 ng) DNA, then completed to volume (approx. 16.1 µl) with ultra-pure water. PCR amplifications were conducted under the following protocol: 94 °C × 3 min + 30 cycles (94 °C × 1 min + 60.5 °C × 1 min + 72 °C × 2 min) + 72 °C × 7 min. Primers used were 18S-IGS (5’-GAGACAAGCATATGACTACTGGCAGGATCAACCAG-3’) and 26S-IGS (5’-GGATTGTTCACCCACCAATAGGGAACGTGAGCTG-3’) (Baldwin and Markos 1998).

The quality of the PCR products was evaluated by agarose electrophoresis (3 μl of final volume plus 2 μl of bromophenol blue, gel prepared with 0.5× TBE and 1% agarose, run at 120 volts and 25 amperes 30 min). PCR products were sequenced at Macrogen (http://www.macrogen.com/eng/) using the same amplification primers. The sequencing products were assembled and edited using the Sequencher v. 5.2.3. An initial automated alignment was conducted with MAFFT (Katoh et al. 2002) using the E-INS-i algorithm option, a 100PAM/k = 2 scoring matrix, a gap opening penalty of 1.3, and an offset value of 0.123. The alignments were visually inspected and manually edited for further improvement. The Akaike Information Criterion (AIC), implemented in jModeltest (Posada 2008) was used to select the best model of nucleotide substitution for each alignment. The selected models were TVM+I+G for ETS and GTR+I+G for ITS. Phylogenetic analyses were performed with MrBayes v.3.2.5 (Ronquist et al. 2012) separately for each dataset, and subsequently concatenated with each partition treated as independent and associated with its own evolutionary model. Analyses were performed using default parameters for 5 million generations. Two independent threads were run. Convergence was assessed with both MrBayes and Tracer (Rambaut et al. 2018). Posterior Probabilities (PP) ≤ 0.95 were considered weakly supported whereas PP of 0.95–1.0 were deemed strongly supported (Alfaro et al. 2003).

To examine the evolution of fruit types within New World *Albizia* we utilize a phylogeny derived from a new analysis based on data of Ringelberg et al. (2022) for the Jupunba clade, as described in Soares et al. (2022), because it is based on a large set of 560 nuclear exons and flanking non-coding regions, and therefore shows enhanced resolution within this clade compared to the ITS + ETS phylogeny.

## Results

Alignments of our combined datasets recovered by MAFFT required few manual adjustments. The ETS sequences had 381 bp and, once aligned, 52% of the data were informative. In the case of ITS, the sequences were slightly longer, 551 bp but only 34% were informative.

**Figure 3.**
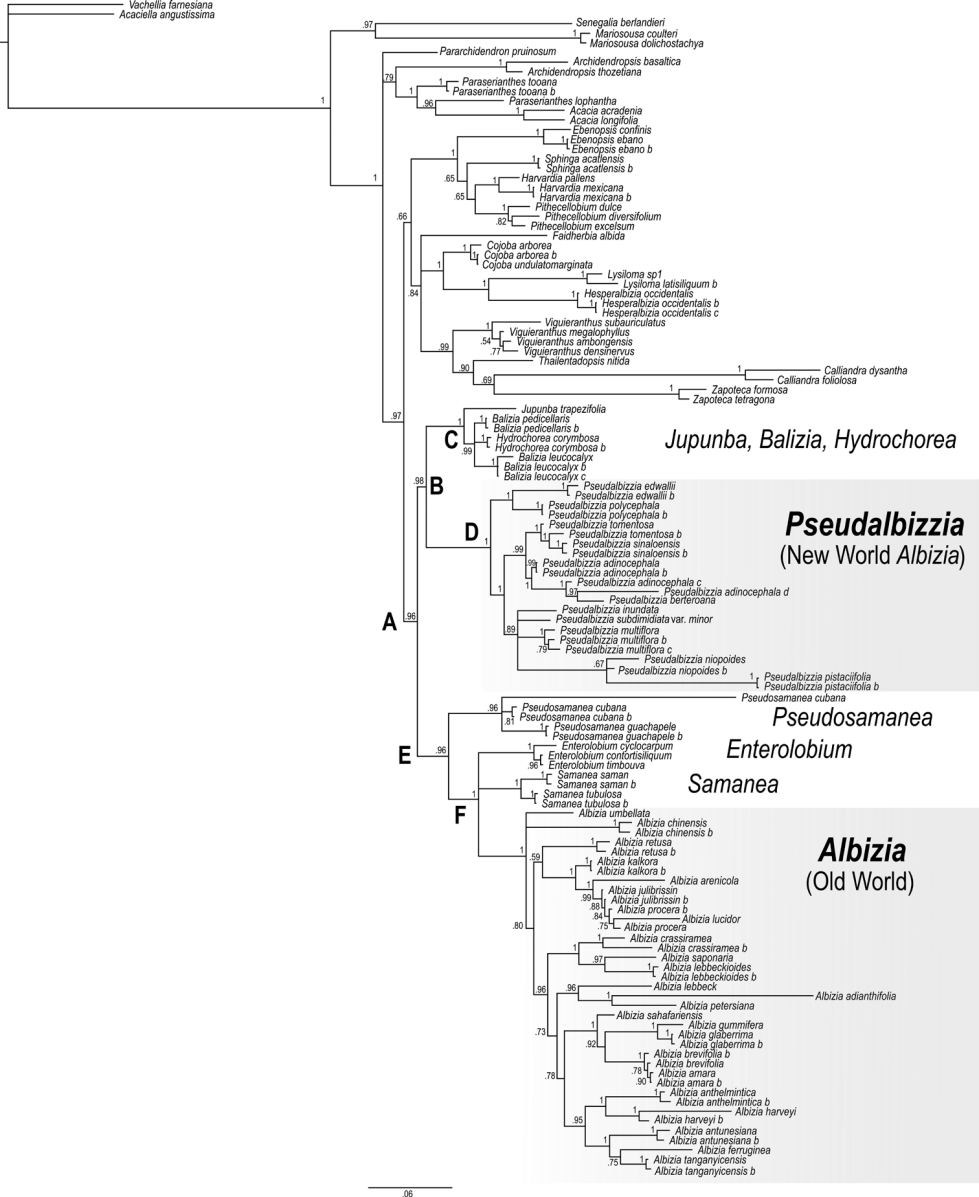
Phylogeny of the ingoid clade (sensu Koenen et al. 2020), i.e., the traditionally recognized tribes Ingeae + Acaciaeae (excl. *Vachellia*). Phylogram derived from Bayesian analysis in MrBayes of the combined ETS and ITS data for *Albizia* and related genera. Main clades are labeled **A–F** (see text). Posterior support values are indicated above branches.

None of the molecular-based analyses (ETS, ITS, and ETS+ITS) using Bayesian inference recovered the genus *Albizia* as monophyletic. The combined ETS + ITS phylogeny (Fig. 3) is used as the basis for discussing the results in detail. The outgroup *Vachelliafarnesiana*, plus *Acaciellaangustissima* (Mill.) Britton & Rose, *Senegaliaberlandieri* (Benth.) Britton & Rose and two species of *Mariosousa* Seigler & Ebinger form a paraphyletic grade subtending a fully supported clade (PP = 1) that includes all members of the tribe Ingeae as well as *Acacia* s.s.

The most relevant clade from the perspective of this study is highly supported (PP = 0.96) and includes all members of the genus *Albizia* and a few other genera of tribe Ingeae (clade A). The genus *Albizia*, as currently circumscribed, is non-monophyletic with species placed in two separate, strongly supported clades (Fig. 3). As in Koenen et al. (2020), species of Albiziasect.Arthrosamanea are placed in clade B (PP = 0.96), which equates to the Jupunba clade of Koenen et al. (2020). All species that were sampled from this section are included in this clade (Fig. 3 clade D), which received full support (PP = 1), and as in Koenen et al. (2020) this section is sister to clade C (PP = 1) comprising the genera *Jupunba*, *Balizia* and *Hydrochorea*.

**Figure 4.**
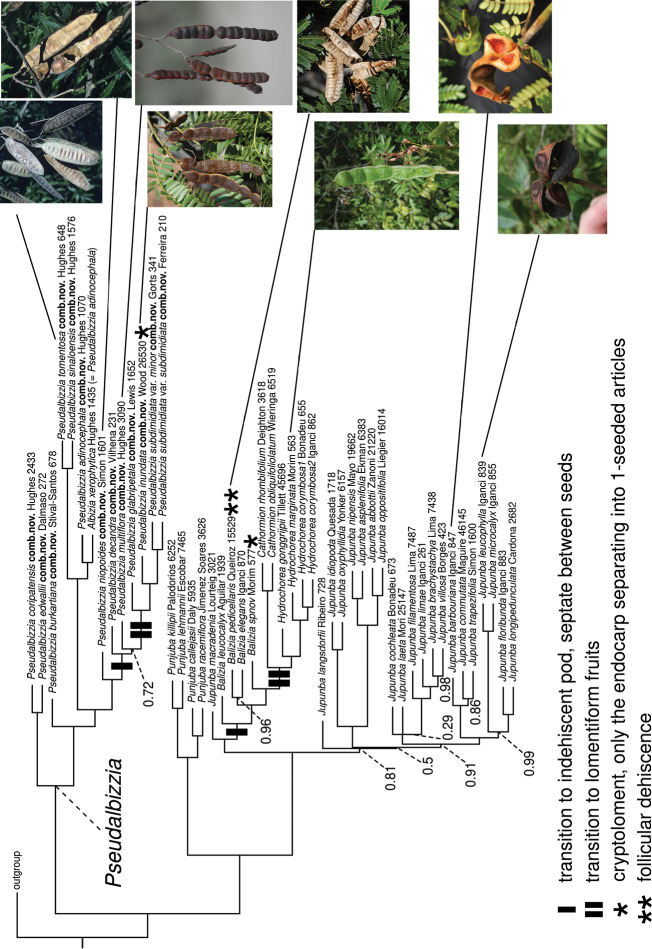
Phylogeny of the Jupunba clade redrawn from an ASTRAL species tree analysis by Soares et al. (2022) that utilizes data from Ringelberg et al. (2022) showing the evolutionary transitions from ancestrally papery, plano-compressed fruits to septate indehiscent fruits and subsequently to lomentiform hydrochorous fruits associated with species growing in seasonally inundated habitats in *Pseudalbizzia* and similar parallel transitions in *Balizia* and *Hydrochorea*. Photos of *Pseudalbizziainundata*, *P.multiflora*, *P.niopoidesP.tomentosa* and *Baliziapedicellaris*, by Colin Hughes, of *Hydrochoreamarginata*, *Jupunbabarbouriana* and *J.leucophylla*, by Erik Koenen.

Within sect. Arthrosamanea, three clades are well supported, one comprising *Albiziapolycephala* (Benth.) Killip ex Record and *Albiziaedwallii* (Hoehne) Barneby & J.W. Grimes of ser. Paniculatae, a second clade comprising species of ser. Paniculatae endemic to Mexico, Central America, and the Caribbean, and a third clade that includes *Albizianiopoides* (Benth.) Burkart (also ser. Paniculatae) and the species from the other three series. The phylogeny of Ringelberg et al. (2022) presented here in Fig. 4 based on a new analysis of the Jupunba clade accessions from Soares et al. (2022), has greater resolution within sect. Arthrosamanea and shows that ser. Paniculatae forms a well-resolved paraphyletic grade in which the other three series are nested. The other two non-monospecific series also appear to be non-monophyletic, with the monospecific ser. Inundatae nested inside ser. Arthrosamanea and these together in turn nested in ser. Multiflorae, although support for the paraphyly of ser. Multiflorae is only 0.72 pp.

The Old World species of *Albizia* form a monophyletic group (PP = 1) placed in clade E (PP = 0.96) (Fig. 3), with the genera *Enterolobium* Mart., *Samanea*, and *Pseudosamanea*. Within clade E *Pseudosamanea* (PP = 0.96) is sister to clade F which includes *Enterolobium*, *Samanea* and Old World *Albizia*. All these clades have high support (PP = 1). These analyses also support the transfer of *Cathormionumbellatum* Kosterm., which is placed in the Old World *Albizia* clade (Fig. 3), to *Albizia*, as proposed by Koenen et al. (2020).

Our analyses also confirm that the monotypic genus *Hesperalbizia*: *H.occidentalis* (Brandegee) Barneby & J.W. Grimes is sister to *Lysiloma*, in the Cojoba clade (sensu Koenen et al. 2020), unrelated to either New World *Albizia* (clade D) or Old World *Albizia* (clade F), as previously shown by Duno de Stefano et al. (2021). Furthermore, *Pseudosamaneaguachapele* (Kunth) Harms (previously *Albiziaguachapele* (Kunth) Dugand in Rico Arce et al. (2008)) is also unrelated to New World *Albizia* but is instead a member of clade E, sister to clade F which includes *Enterolobium*, *Samanea* and Old World *Albizia*.

In both Albiziasect.Arthrosamanea and the closely related *Balizia* and *Hydrochorea*, these phylogenies suggest that lomentiform fruits were independently derived from indehiscent fruits that are septate between the seeds, as species with the latter fruit type form paraphyletic grades to the lomentiform species in both cases (Fig. 4). In turn, these indehiscent septate fruits are nested within paraphyletic assemblages of species with fruits that dehisce along one or both sutures in both groups. Interestingly, in both cases, a single species with crypto-lomentiform fruits is found, but it is not clear whether these were derived from the same ancestral fruit type or not. In *Hydrochorea* this crypto-lomentiform species appears as an intermediate between indehiscent and lomentiform species, while in Albiziasect.Arthrosamanea the crypto-lomentiform-fruited *A.inundata* appears to be derived from a lomentiform-fruited ancestor. Another difference between these two groups is that the follicular dehiscence of *Baliziapedicellaris* (DC.) Barneby & J.W. Grimes fruits appears to be secondarily derived from indehiscent fruits, but we note that similar dehiscence is also found in a few species of *Jupunba*.

## Discussion

This study addresses the non-monophyly of the genus *Albizia* and our results provide important insights into the evolutionary history of the neotropical species placed in sect. Arthrosamanea, with implications for their taxonomic classification. We show that sect. Arthrosamanea, with expanded taxon sampling relative to Koenen et al. (2020), and as also shown by the study of Ringelberg et al. (2022), is monophyletic with only two exceptions: *Albizialeonardii* Barneby & J.W. Grimes which is placed among taxa of the ‘senegalioid grade’ (Ringelberg et al. 2022; Terra et al. 2022) and *Albiziacarbonaria* Britton that is more closely related to *Pseudosamanea* (Koenen 2022b; Ringelberg et al. 2022).

The geographically-based splitting of a large genus in tribe Ingeae, such as *Albizia*, which occupies a pantropical distribution, is not unprecedented, nor unexpected, especially given the lack of pantropical monographic synthesis or geographically widely sampled phylogenies for the mimosoid clade. For example, the genus *Pithecellobium*, once the largest genus of tribe Ingeae, has been progressively divided during the last 50 years into multiple genera (see Brown et al. 2008 for a general history of the tribe). Another example is the genus *Calliandra* Benth. for which the New World species of *Calliandra*. ser. Laetevirentes were segregated into *Zapoteca* H.M. Hern. (Hernández 1986), and almost all Old World species allocated progressively to other segregated genera: *Viguieranthus* Villiers (Villiers 2002), *Thailentadopsis* Kosterm. (Lewis and Schrire 2003), *Sanjappa* E.R. Souza & M.V. Krishnaraj (Souza et al. 2016) and *Afrocalliandra* E.R. Souza & L.P. Queiroz (Souza et al. 2013). All these taxonomic rearrangements were supported by morpho-anatomical and molecular phylogenetic analyses. Molecular data have also demonstrated that *Abarema* is polyphyletic (Iganci et al. 2015), prompting reinstatement of the genera *Jupunba* and *Punjuba* Britton & Rose (Soares et al. 2021). Finally, neither *Zygia* P. Browne nor *Marmaroxylon* Killip are monophyletic, although a new generic classification for those genera has yet to be proposed (Ferm et al. 2019). The non-monophyly of *Albizia* documented here and elsewhere (Koenen et al. 2020; Ringelberg et al. 2022) is thus not a surprise and reflects the state of flux surrounding generic delimitation in mimosoids, especially within the ingoid clade.

Some of the taxonomic proposals of Barneby and Grimes (1996) relative to the American segregates of *Albizia* s.l. are confirmed by our results. The genera *Balizia* and *Hydrochorea* form part of the Jupunba clade (sensu Koenen et al. 2020) (Figs 3 and 4) (Iganci et al. 2015), although neither *Hydrochorea* nor *Balizia* are monophyletic in our phylogeny (Figs 3 and 4, see Soares et al. 2022). Two neotropical species, included in *Albizia* by Rico Arce et al. (2008) are also placed outside New World *Albizia* (Fig. 3): *Hesperalbiziaoccidentalis* is closely related to *Lysiloma*, in agreement with previous results (Iganci et al. 2015; Duno de Stefano et al. 2021); similarly, *Pseudosamaneaguachapele*, is also placed outside *Albizia* in our phylogeny, emerging, as expected, together with the other species *Pseudosamaneacubana* (Britton & Rose) Barneby & J.W. Grimes, although relationships within this clade are unresolved (Fig. 3).

Here we show that the dehiscent, papery, plano-compressed fruit type is ancestral within Albiziasect.Arthrosamanea (Fig. 4) and is associated with species growing predominantly in seasonally dry tropical forest and woodland, with successive shifts to septate indehiscent fruits and then lomentiform fruits with hydrochorous seed dispersal associated with species growing in seasonally inundated varzea forest, riverine habitats and low-lying margins of palm and white-sand savannas (Fig. 4). Interestingly, in the sister group of Albiziasect.Arthrosamanea, the mainly neotropical clade composed of *Jupunba*, *Punjuba*, *Balizia*, and *Hydrochorea*, a similar parallel evolutionary transition in fruit types is apparent. In *Jupunba* and *Punjuba*, fruits are always dehiscent, while a transition to septate indehiscent fruits occurred in *Balizia*, an exception being *Baliziapedicellaris* which has follicular dehiscence and a newly described species with crypto-lomentiform fruits (Fig. 4, and Soares et al. 2022). Nested within the paraphyletic *Balizia* is a clade comprising the genus *Hydrochorea* plus two African species of *Cathormion*, all species of which have indehiscent lomentiform fruits adapted for hydrochory and are found in riparian or other periodically flooded habitats in the Amazon basin, West Africa and the Congo basin (Fig. 4, and Soares et al. 2022).

Barneby and Grimes (1996) pointed out that a radiation of species with similarly heterogeneous fruit types to that seen in section Arthrosamanea occurs in Madagascan *Albizia* s.s. and that the association between lomentiform fruits, hydrochorous seed dispersal, and seasonally flooded habitats is also apparent in Old World *Albizia* s.s. For example, *Albiziadolichadena*, *A.moniliformis*, *A.rosulata*, and *A.umbellata* from Australasia also have lomentiform fruits and are distributed near streams or in riparian and swamp forests (Rico Arce et al. 2008). Furthermore, as indicated above and pointed out by Barneby and Grimes (1996), similar transitions to lomentiform fruits have occurred in parallel in several other lineages across the ingoid clade, including *Cathormionaltissimum* (Hook.f.) Hutch. & Dandy (sometimes referred to as *Albiziaaltissima* Hook.f.; Koenen 2022a) and *Senegaliarostrata* (Humb. & Bonpl. ex Willd.) Seigler & Ebinger (syn. *Dugandiarostrata* (Humb. & Bonpl. ex Willd.) Britton & Killip, syn. *Manganaroaarticulata* Speg.; Barneby and Grimes 1996: 204) in all cases apparently also closely associated with riparian and/or periodically inundated habitats. These repeated parallel derivations of similar, but not strictly homologous fruit types attest to the high evolvability of the mimosoid fruit more generally. In the light of phylogenetic evidence, it is now clear that these evolutionarily highly labile morphological adaptations of the fruit related to seed dispersal syndrome do not provide reliable characters for generic delimitation, supporting inclusion of the species that were placed in ser. Arthrosamanea, ser. Inundatae and ser. Multiflorae within Albiziasect.Arthrosamanea by Barneby and Grimes (1996), i.e., the clade of New World *Albizia* that is recovered in our analysis.

### Taxonomic treatment

There are two validly published generic names – *Pseudalbizzia* of Britton and Rose (1928) and *Arthrosamanea* of Britton and Killip (1936) – that could be applied to the New World clade of *Albizia*. In accordance with Principle III of the International Code of Nomenclature (Turland et al. 2018), we reinstate *Pseudalbizzia*, the earlier name associated with this clade, and provide the corresponding new combinations for its constituent species.

#### Pseudalbizzia

Taxon classificationPlantaeFabalesFabaceae

Britton & Rose, N. Am. Fl. 23: 48. 1928.

2CAC816E-545E-5599-8431-91F617C81151

##### Type.

*Pseudalbizziaberteroana* Britton & Rose.

*Arthrosamanea* Britton & Rose, in Britton & Killip, Ann. New York Acad. Sci. 35: 128, 1936. AlbiziasectionArthrosamanea (Britton & Rose) Barneby & J.W. Grimes, Mem. New York Bot. Gard. 74(1): 206. 1996. Type: *Arthrosamaneapistaciifolia* Britton & Rose.

##### Description.

Unarmed ***trees*** with sympodial growth, up to 30 m, rarely small treelets of c. 3 m, microphyllidious to macrophyllidious; trunk 35–120(–150) cm dbh; young stems and all leaves and inflorescence-axes more or less densely tomentellous to pilosulous; stipules puberulent to glabrous, deltate, narrowly triangular, triangular-ovate, narrowly ovate, or narrowly lanceolate, veinless or faintly 3-veined, falling early to tardily, perhaps sometimes obsolete and/or lacking on mature leaves. ***Leaves*** bipinnate, not sensitive, (1–)2–15(–19) pairs of pinnae; leaflets (2–)16–52(–63) pairs per pinna; a nectary immediately below first pair of pinnae, near or well below mid-petiole, sometimes lacking or reduced to a minute pore, round, elliptic or vertically elongate, either shallow-cupular or almost plane, thick-rimmed, sometimes immersed in petiolar groove or even obsolete, much smaller nectaries at some distal pinnae, at the tip of most pinnae, and between 1–2 furthest pairs of leaflets; leaflets gently decrescent toward each end of the rachis or toward the base of the rachis or sub-equilong, the first pair of leaflets often reduced to paraphyllidia, sometimes minute, sometimes absent or perhaps falling early, the blades of the remaining leaflets elliptic, elliptic-ovate, oblong-elliptic, narrowly oblong-elliptic, lance-oblong to linear-lanceolate, base obliquely truncate to shallowly semi-cordate, apex deltately subacute, deltately acute to subacute, obtuse or apiculate, the larger ones (1.5–)2–4(–6) times as long as wide, margin strongly to slightly revolute; venation generally palmate, of 2–4(–5) veins from the pulvinule, the nearly straight main vein a little forwardly displaced and giving rise on each side to 2–13 major secondary veins, the inner of 2(–3) posterior primary veins incurved-ascending to anastomose slightly beyond mid-blade, the outer posterior vein and sometimes a faint anterior one very short and weak, all venation immersed on upper face. ***Inflorescence*** primary axis up to 30 cm long; peduncles (1–)2–8(–10) per node of the capitulate or corymbose-umbellate inflorescence, capitula 8–26(–40)-flowered; bracts heteromorphic or homomorphic, ovate, oblong-obovate or spatulate, linear-spatulate, falling early or persistent, sessile or shortly pedicellate, the flowers moderately to strongly dimorphic, the terminal ones generally longer. ***Flowers*** 5-merous, rarely 6-merous, glabrous to densely pubescent externally. Peripheral flowers: calyx campanulate, turbinate, turbinate-campanulate or narrowly campanulate, sessile or short pedicellate, lobes very short, depressed-deltate, ovate or triangular, glabrous or puberulent; corolla narrowly trumpet-shaped, erect or recurved, lobes ovate to lance-ovate; androecium with 9–30(–32) stamens, up to 20 mm long, united at the base forming a clear stemonozone, the staminal tube as long or longer than the stemonozone; ovary sessile or shortly stipitate, slenderly ellipsoid, conical at apex, glabrous or pubescent; style a little longer than the stamens, slightly dilated at the stigma. Terminal flowers: sessile or almost so, calyx shallowly campanulate to broadly campanulate, corolla tubular; androecium with 16–38(–42) stamens, 8.5–11.5(–13) mm long, united at the base forming a clear stemonozone, staminal tube equalling or longer than the stemonozone. ***Fruits*** solitary, or rarely 2–4 per capitulum, sessile, subsessile or cuneately contracted at base into a short pseudo-stipe, the body linear, linear-elliptic, narrowly elliptic-oblong, straight or nearly straight, sometimes decurved, plano-compressed, apex rounded but minutely apiculate to obtuse, (8–)13(–15)-seeded; valves papery, coriaceous, or grossly ligneous, olivaceous, castaneous, fuscous-greenish, or brown becoming tan-brown, closely transverse venulose, minutely puberulous, tomentulose, glabrescent to glabrous, framed by straight sutures or dilated, sometimes 3-angulate but not winged, transversely or horizontally, dehiscence tardy to very tardy, inert, through both sutures or dehiscence 0, in the latter, the pod crypto-lomentiform, incipiently lomentiform or lomentiform, then the whole fruit long persistent on the tree, commonly falling entire and breaking on the ground into 8–12 individually indehiscent segments, funicle apically sigmoid or ribbon-like (not sigmoid), lentiform; ***seeds*** obliquely ascending or straight, disciform, oblong-ellipsoid, elliptic, strongly compressed, the translucent, brownish or greyish testa produced as a peripheral wing, adherent to the embryo, which does not fill the testa-cavity, the pleurogram small, inversely U-shaped or U-shaped.

##### Notes.

The genus forms a group that is homogeneous in most respects, but diverse in the late developmental stages of the fruit, including: 1) fruit opening type: dehiscent, indehiscent, or irregularly breaking, 2) lateral shape: flat to conspicuously raised over the seed chambers, 3) texture and consistency of the valves: papery, chartaceous to woody (Barneby and Grimes 1996). Figs 1, 2 and 4.

*Pseudalbizzia* (clade D) is the sister group of the *Jupunba*-*Punjuba*-*Balizia*-*Hydrochorea* clade (Fig. 3). *Jupunba* and *Punjuba* are markedly different morphologically, having spirally twisted dehiscent fruits with a red or ochre endocarp, reminiscent of the fruits of several other genera in tribe Ingeae (e.g., some *Pithecellobium* species, and some species of *Archidendron* F. Muell. and *Cojoba* Britton & Rose). The red or red-brown testa of the seeds of *Jupunba* and *Punjuba* are very distinctive, and are never black, and the embryo is nearly always aniline-blue due to the presence of delphinidin (an anthocyanidin). *Punjuba* is furthermore distinguished by its spicate inflorescences, which are not seen in *Pseudalbizzia*. *Balizia* has ligneous, indehiscent or tardily dehiscent pods, their seeds being released sometimes only after decay of the valves on the floor of terra firme forest, whereas in *Hydrochorea* the fruits are lomentiform, adapted to dispersal by water. The fruits of *Hydrochorea* recall some species of *Pseudalbizzia* adapted to similar riparian habitats. However, the species of *Pseudalbizzia* are markedly different in form of inflorescence, leaflet-venation, and shape of the ovary.

Two species previously placed in *Albizia* from the New World which were not included in our phylogenetic analysis, *Albiziacarbonaria* and *A.leonardii*, have since been shown to be placed outside the New World *Albizia* clade (Ringelberg et al. 2022; Koenen 2022b; Terra et al. 2022). Two other species, also not sampled here, nor by Ringelberg et al. (2022), are here tentatively included in *Pseudalbizzia*: *Albiziabarinensis* L. Cárdenas and *Albiziabuntingii* Barneby & J.W. Grimes (see below for discussion about the placement of these species). The genus *Pseudalbizzia* was published in the Flora of North America (Britton and Rose 1928) and included just a single species, *P.berteroana*. The original description of *Pseudalbizzia* closely matches *Albizia* and no characters distinguishing the two genera were discussed by Britton and Rose (1928). The generic name *Arthrosamanea* was also published by Britton & Rose, again with a single species, *A.pistaciifolia* (Willd.) Britton & Rose, in an account of the Mimosaceae and Caesalpiniaceae of Colombia (Britton and Killip 1936), but again no differences between the genus and *Albizia* or *Pseudalbizzia* were mentioned.

**Figure 5.**
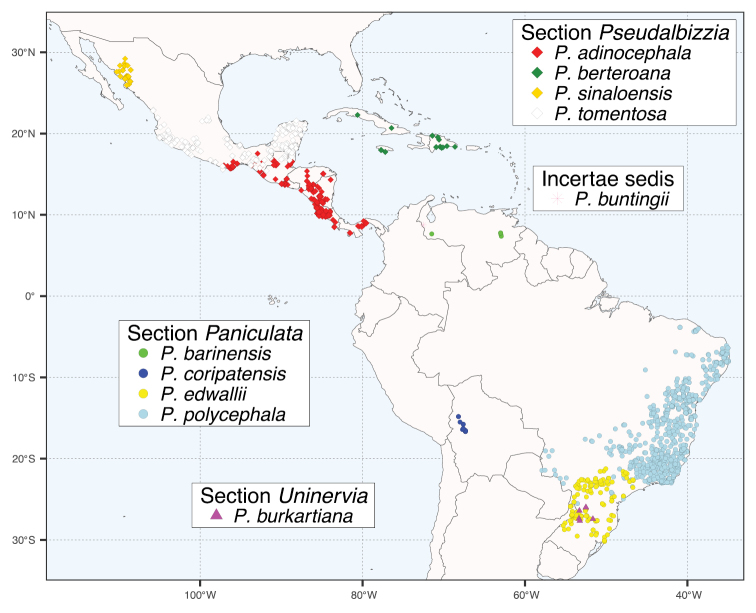
Distribution map of *Pseudalbizzia* sections *Paniculata*, *Pseudalbizzia*, *Uninervia* and *Pseudalbizziabuntingii* (incertae sedis), as per the legend.

**Figure 6.**
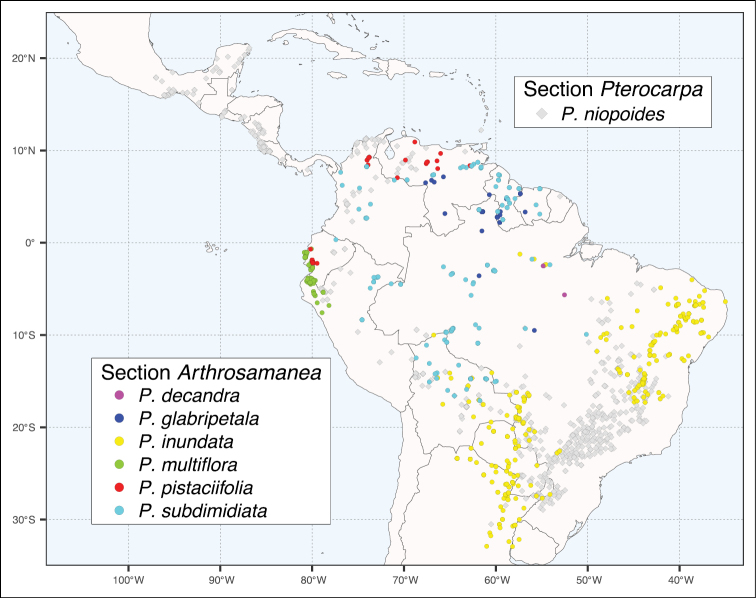
Distribution map of *Pseudalbizzia* sections *Arthrosamanea* and *Pterocarpa*, as per the legend.

*Pseudalbizzia* as circumscribed here comprises 17 species and 5 varieties ranging in distribution from northwestern Mexico to northern Argentina and including the Greater Antilles (Figs 5 and 6). Full synonymy, detailed species descriptions, geographical distributions, representative samples of all species and keys for their identification can be found (under the name *Albizia*) in Barneby and Grimes (1996), Linares (2005) and Rico Arce et al. (2008). Finally, we propose a new sectional classification of *Pseudalbizzia* to account for the non-monophyly of the series of Barneby and Grimes (1996), based on the phylogenies (Figs 3 and 4) which sampled nearly all species. A key to the sections is provided.

### Key to the sections of the genus *Pseudalbizzia*

**Table:** 

1	Leaflets with a single vein from the pulvinule	**sect. Uninervia**
–	Leaflets with 3–5 veins from the pulvinule	**2**
2	Fruits with a narrowly winged margin, seeds oblique, foliage microphyllidious	**sect. Pterocarpa**
–	Fruit margins not winged, or if winged, then foliage macrophyllidious and seeds straight	**3**
3	Fruits indehiscent and septate or lomentiform	** sect. Arthrosamanea**
–	Fruits dehiscent, plano-compressed, valves papery, not septate	**4**
4	Micro- to mesophyllidious foliage, distributed in South America	**sect. Paniculata**
–	Macro- or microphyllidious foliage, distributed in Mexico, Central America and the Caribbean	**sect. Pseudalbizzia**

#### Pseudalbizzia sect. Paniculatae

Taxon classificationPlantaeFabalesFabaceae

(Benth.) E.J.M. Koenen & Duno stat. nov. and sect. nov.

51C280F7-9E14-54A5-A99D-9D3E63CC69FE

urn:lsid:ipni.org:names:77303802-1

Pithecellobium sect. Samanea ser. Paniculatae Benth. pro parte, London J. Bot. 3: 219. 1844.
Albizia sect. Arthrosamanea ser. Paniculatae (Benth.) Barneby & J.W. Grimes pro parte, Mem. New York Bot. Gard. 74(1): 208. 1996. Type species (designated by Barneby and Grimes, Mem. New York Bot. Gard. 74(1): 208. 1996.): Pithecellobiumpolycephalum Benth. = Pseudalbizziapolycephala (Benth.) E.J.M. Koenen & Duno.
Pithecellobium sect. Samanea ser. Parviflorae [sic] Benth. pro parte, Trans. Linn. Soc. London 30: 591 (exclus. sp. 77). 1875 & in Martius, Fl. Bras. 15(2): 445. 1876. Type species (designated by Barneby and Grimes, Mem. New York Bot. Gard. 74(1): 208. 1996.): Pithecellobiumpolycephalum Benth. = Pseudalbizziapolycephala (Benth.) E.J.M. Koenen & Duno.

##### Type.

*Pithecellobiumpolycephalum* Benth. = *Pseudalbizziapolycephala* (Benth.) E.J.M. Koenen & Duno.

##### Notes.

Micro- to mesophyllidious trees with paniculate compound inflorescences of efoliate pseudoracemes and dehiscent plano-compressed papery fruits. Four species of humid, semi-deciduous and seasonally dry tropical and extratropical forests and woodland in South America (Fig. 5).

#### Pseudalbizzia barinensis

Taxon classificationPlantaeFabalesFabaceae

(L. Cárdenas) E.J.M. Koenen & Duno comb. nov.

69D7200D-E6A5-5EC2-9482-4A99AA5F26F6

urn:lsid:ipni.org:names:77303803-1

##### Basionym.

*Albiziabarinensis* L. Cárdenas, Ernstia 21: 5, f. sn. 1983.

##### Type.

Venezuela. Barinas, muy cerca de Punta de Piedra, 3 Apr 1976, *L. Cardenas de Guevara 2273* (holotype: MY; isotypes: BM!, F! [F0093839F], K! [K000527984], NY! [NY00001781], RB! [RB00539860], US! [US00385615], VEN).

##### Notes.

This species has not been included in any phylogenetic analysis, but its foliage, efoliate pseudoracemes and plano-compressed papery fruits leave little doubt that it should be placed in *Pseudalbizzia*. It is here included in section Paniculata based on these characters and its South American distribution.

#### Pseudalbizzia coripatensis

Taxon classificationPlantaeFabalesFabaceae

(Rusby) E.J.M. Koenen & Duno comb. nov.

0B73E401-51FE-5679-8C68-8B4BD54CB84E

urn:lsid:ipni.org:names:77303804-1

##### Basionym.

*Pithecellobiumcoripatense* Rusby, Bull. New York Bot. Gard. 4: 349. 1907.

##### Type.

Bolivia. La Paz, Sur Yungas, at Coripata, 6 May 1894, *M. Bang 2176* (holotype: NY! [NY00334642]; isotypes: BM! [BM000952433], G-2! [G00364414, G00364429], GH-2! [GH00064010, GH00064011], M! [M0218258], K! [K000527985], MINN, MO! [MO-954213], US).

#### Pseudalbizzia edwallii

Taxon classificationPlantaeFabalesFabaceae

(Hoehne) E.J.M. Koenen & Duno comb. nov.

698DB3B9-86E0-5B91-9745-37BB74A055DB

urn:lsid:ipni.org:names:77303805-1

##### Basionym.

*Pithecellobiumedwallii* Hoehne, Bol. Inst. Brasil. Sci. 2: 243. 1926.

##### Type.

Brazil, São Paulo, *G. Edwall 5608* (lectotype: SP, designated by Barneby and Grimes, Mem. New York Bot. Gard. 74(1): 209. 1996).

#### Pseudalbizzia polycephala

Taxon classificationPlantaeFabalesFabaceae

(Benth.) E.J.M. Koenen & Duno comb. nov.

AFACA64D-2A08-57CE-87AE-F8501CC2B269

urn:lsid:ipni.org:names:77303806-1

##### Basionym.

*Pithecellobiumpolycephalum* Benth., London J. Bot. 3: 219. 1844.

##### Type.

Brazil. Rio de Janeiro, *J.B.E. Pohl 1420* (lectotype: K! (*herb. Bentham*) [K000528000], designated by Barneby and Grimes, Mem. New York Bot. Gard. 74(1): 208. 1996).

#### Pseudalbizzia sect. Uninervia

Taxon classificationPlantaeFabalesFabaceae

E.J.M. Koenen & Duno sect. nov.

85763601-3140-5932-A621-CC4521B16C7E

urn:lsid:ipni.org:names:77303807-1

##### Type.

*Albiziaburkartiana* Barneby & J.W. Grimes = *Pseudalbizziaburkartiana* (Barneby & J.W. Grimes) E.J.M. Koenen & Duno.

##### Notes.

Microphyllidious trees with the inflorescences of section Paniculata, but with a single vein from the pulvinule at the base of the leaflets. A single, narrowly endemic species in Paraná pine woodland and the Southern Mata Atlantica of Brazil (Fig. 5).

#### Pseudalbizzia burkartiana

Taxon classificationPlantaeFabalesFabaceae

(Barneby & J.W. Grimes) E.J.M. Koenen & Duno comb. nov.

7750A1A4-3BC0-5644-BEA1-054594AA514E

urn:lsid:ipni.org:names:77303808-1

##### Basionym.

*Albiziaburkartiana* Barneby & J.W. Grimes, Mem. New York Bot. Gard. 74(1): 211–212. 1996.

##### Type.

Brazil. Santa Catarina, Capinzal, on upper Rio Uruguai, 700 m, 21 Dec 1973, *P.R. Reitz & R. M. Klein 14359* (holotype: NY! [NY00001783]; isotype: US! [US00811452]).

##### Notes.

In the protologue the fruits were not described as these were not known at that time. This rare, locally endemic species has since been collected in fruit (*Stival-Santos 678*, BR), and we here provide a description of these. Fruits sessile but with a narrow pseudo-stipitate base, dehiscent along both slightly thickened sutures, the valves plano-compressed, papery in texture, light brown with finely prominent transverse veins, 6.5–12 × 1.2–1.6 cm, 7–12-seeded when well-fertilized.

#### Pseudalbizzia sect. Pseudalbizzia

Taxon classificationPlantaeFabalesFabaceae

.

5BE01C55-1AF6-500C-90AC-7AFE6176F62F

##### Notes.

Trees with micro- or macrophyllidious foliage, inflorescences composed of efoliate pseudoracemes arising singly from a leaf axil or sometimes the capitula solitary or paired in the leaf axils, or the pseudoracemes combined into a terminal panicle, fruits plano-compressed with papery valves, dehiscent along both sutures or more rarely indehiscent (in *P.berteroana*), sometimes with a winged margin, seeds straight. Four species predominantly of seasonally dry tropical forests in Mexico, Central America and the Caribbean (Fig. 5).

#### Pseudalbizzia adinocephala

Taxon classificationPlantaeFabalesFabaceae

(Donn. Sm.) E.J.M. Koenen & Duno comb. nov.

9481FEBE-D8DF-5829-935C-B582EED8281A

urn:lsid:ipni.org:names:77303809-1

Albizia xerophytica J. Linares, syn. nov., Revista Mex. Biodiversidad 76: 7. 2005. Type: Honduras. El Paraíso, Municipio Morocelí, orillas de Quebrada Grande c. 3.9 km al NE de Morocelí por el camino hacia El Plan. 2002. *J.L. Linares et al. 5674* (holotype: MEXU! [MEXU01160777]; isotype: EAP).

##### Basionym.

*Pithecellobiumadinocephalum* Donn. Sm., Bot. Gaz. Crawfordsville. 57: 419. 1914.

##### Type.

Costa Rica. San José, Ad fundum La Verbena prope Alajuelita, 100 m, Aug 1894, *A. Tonduz 8932* (US-3); Dec 1894 (lectotype: *A. Tonduz* 9077 [US-212774]!; isolectotypes: BR-3! [BR0000005189519, BR0000005189182, BR0000005189847], G! [G00364416], designated by Barneby and Grimes, Mem. New York Bot. Gard. 74(1): 218. 1996).

##### Notes.

*Albiziaxerophytica* was described from material from dry forest habitats in southern Honduras based on minor differences in leaf and fruit morphology, but we do not consider these to be significantly different from the range of variation that is observed in *P.adinocephala* and prefer the broader concept of the species as described in Barneby and Grimes (1996: 218–220). The difference in habitat (i.e., lower rainfall regions) also appears to be minor, as some specimens from wetter sites have been identified as *A.xerophytica* (see map in Rico Arce et al. 2008) while specimens of *P.adinocephala* have been collected across the full range of drier and wetter sites. Finally, the distribution of *A.xerophytica* is entirely enclosed by the much wider range of *P.adinocephala*.

#### Pseudalbizzia berteroana

Taxon classificationPlantaeFabalesFabaceae

(Balb. ex DC.) Britton & Rose, N. Amer. Fl. 23: 48. 1928.

8DFA2951-ABC6-58DB-9606-6EC6DDEDD7D4

##### Basionym.

*Acaciaberteroana* Balb. ex DC., Prodr. 2: 470. 1825.

##### Type.

Republica Dominicana, Sto. Domingo, *C.L.G. Bertero, herb. Balbis s.n.*, 1821 (holotype: G; isotype: M! [M0218254]).

#### Pseudalbizzia sinaloensis

Taxon classificationPlantaeFabalesFabaceae

(Britton & Rose) E.J.M. Koenen & Duno comb. nov.

A29E728A-2240-5690-BD6D-285826FC85EE

urn:lsid:ipni.org:names:77303810-1

##### Basionym.

*Albizia sinaloënsis* in Britton & Rose, N. Amer. Fl. 23(1): 45. 1928.

##### Type.

Mexico. Sinaloa, vicinity of Fuerte, 26 March 1910, *J.N. Rose, P.C. Standley & Russell 13559* (holotype: NY! [NY00001775]; isotype: US! [US00000483]).

#### Pseudalbizzia tomentosa

Taxon classificationPlantaeFabalesFabaceae

(M. Micheli) E.J.M. Koenen & Duno comb. nov.

40BDE739-729D-5963-8ACA-852EF9E64D0D

urn:lsid:ipni.org:names:77303811-1

##### Basionym.

*Pithecellobiumtomentosum* M. Micheli, Mém. Soc. Phys. Genève 34: 285, t. 28. 1903.

##### Type.

Mexico. Michoacán, rives de l’Espiritu Santo, 600 m, 19 April 1898 [*E. Langlassé*] *107* (G): Zilmatango, 30 m, aout 1898, n *280* (G). (lectotype: *E. Langlassé 107* G-385667!; isolectotypes: K! [K000082098], NY (fragm.)! [NY00001777], designated by Standley, Contr. U.S. Natl. Herb. 23: 396. 1922).

#### Pseudalbizzia tomentosa var. nayaritensis

Taxon classificationPlantaeFabalesFabaceae

(Britton & Rose) E.J.M. Koenen & Duno comb. nov.

31D62C93-B409-5602-9EDB-FB676F83886F

urn:lsid:ipni.org:names:77303812-1

##### Basionym.

*Albizzianayaritensis* Britton & Rose, N. Amer. Fl. 23: 47. 1928.

##### Type.

Mexico. Nayarit; San Blas, La Palma, 20 m, 1923, *J. González Ortega 90N* (holotype: US! [US00918691]; isotypes: K! [K000082100], NY-2! [NY00001768, NY00001769]).

#### Pseudalbizzia tomentosa var. purpusii

Taxon classificationPlantaeFabalesFabaceae

(Britton & Rose) E.J.M. Koenen & Duno comb. nov.

081C2877-AA81-5327-8F84-B18849331B3E

urn:lsid:ipni.org:names:77303813-1

##### Basionym.

*Albizziapurpusii* Britton & Rose, N. Amer. Fl. 23: 45. 1928.

##### Type.

Mexico. Veracruz, Rancho Remudadero, 19°15'N, 96°34'W, April 1922, *C.A. Purpus 8723* (holotype: NY! [NY00001773]; isotypes: GH! [GH00069252], MO! [MO-120564], UC! [UC214372], US! [US00000479]).

**Pseudalbizziatomentosavar.tomentosa**

#### Pseudalbizzia sect. Pterocarpa

Taxon classificationPlantaeFabalesFabaceae

E.J.M. Koenen & Duno sect. nov.

64902C4B-17E1-5620-9281-ADD482E16FC1

urn:lsid:ipni.org:names:77303814-1

##### Type.

*Pithecellobiumniopoides* Spruce ex Benth. = *Pseudalbizzianiopoides* (Spruce ex Benth.) E.J.M. Koenen & Duno.

##### Notes.

Microphyllidious trees with the inflorescence usually composed of axillary efoliate pseudoracemes, sometimes a partly or wholly terminal panicle (but not surpassing the foliage), the fruit with a narrowly winged margin and seeds oblique. A single widespread species found in deciduous seasonally dry forests, gallery forest, and evergreen forests in Mexico, Central and South America (Fig. 6).

#### Pseudalbizzia niopoides

Taxon classificationPlantaeFabalesFabaceae

(Spruce ex Benth.) E.J.M. Koenen & Duno comb. nov.

E1372BC2-152D-560D-B5A1-10A3BF1297C0

urn:lsid:ipni.org:names:77303815-1

##### Basionym.

*Pithecellobiumniopoides* Spruce ex Benth., Trans. Linn. Soc. London 30: 591. 1875.

##### Type.

Brazil, Pará, Santarem, Nov 1851, *R. Spruce 1088*, *Herb. Bentham* (holotype: K! [K000528013]).

#### Pseudalbizzia niopoides var. colombiana

Taxon classificationPlantaeFabalesFabaceae

(Britton) E.J.M. Koenen & Duno comb. nov.

3C0828F6-F254-508D-8F6C-B1C8EB40D791

urn:lsid:ipni.org:names:77303816-1

Albizia niopoides var. colombiana (Britton) Barneby & J.W. Grimes, Mem. New York Bot. Gard. 74(1): 222. 1996.

##### Basionym.

*Albizziacolombiana* Britton, in Britton & Killip, Ann. New York Acad. Sci. 35: 131. 1936.

##### Type.

Colombia. Magdalena, near Bonda, Santa Marta, 3 August 1899, *H.H. Smith 38* (holotype: NY! [NY00001784]; isotypes: BR! [BR0000005111176], E! [E00313853], K! [K000527990], NY!, U-2! [U0003354, U1253389]).

**Pseudalbizzianiopoidesvar.niopoides**

#### Pseudalbizzia sect. Arthrosamanea

Taxon classificationPlantaeFabalesFabaceae

(Britton & Rose) E.J.M. Koenen & Duno comb. nov.

F83EDACB-A757-557A-8FFE-7B78995BC5BA

urn:lsid:ipni.org:names:77303817-1

Arthrosamanea Britton & Rose, Ann. New York Acad. Sci. 35: 128, pro gen. 1936, *sensu stricto*.Albiziasect.Arthrosamanea (Britton & Rose) Barneby & J.W. Grimes pro parte, Mem. New York Bot. Gard. 74(1): 206. 1996. Type species: Arthrosamaneapistaciifolia (Willd.) Britton & Rose = Mimosapistaciifolia Willd. = Pseudalbizziapistaciifolia (Willd.) E.J.M. Koenen & Duno.
Albizia sect. Arthrosamanea ser. Multiflorae Barneby & J.W. Grimes, Mem. New York Bot. Gard. 74(1): 234. 1996.
Albizia sect. Arthrosamanea ser. Inundatae Barneby & J.W. Grimes, Mem. New York Bot. Gard. 74(1): 238. 1996.

##### Notes.

Micro- or macrophyllidious trees, usually the efoliate pseudoracemes arising singly and only rarely arranged in panicles, fruits indehiscent and septate, or lomentiform, one species crypto-lomentiform. Six species of usually humid, often seasonally inundated forest or riparian habitats in South America (Fig. 6).

#### Pseudalbizzia decandra

Taxon classificationPlantaeFabalesFabaceae

(Ducke) E.J.M. Koenen & Duno comb. nov.

C886C9E6-0DD6-554B-8591-77019E4D11BB

urn:lsid:ipni.org:names:77303818-1

##### Basionym.

*Pithecellobiumdecandrum* Ducke, Arch. Jard. Bot. Rio de Janeiro 5: 121. 1930.

##### Type.

Brazil. Pará, habitat in silvis non inundatis civitatis Pará circa Óbidos, *A. Ducke* (Herb. Amaz. Mus. Pará 15.724, et H.J.B.R. 10.174) et loco Serra do Dedal ad lacum Faro, *A. Ducke* (H.J.B.R. 20.198), ubi florebat Januario 1927, *A. Ducke* (lectotype: *A. Ducke 10174* RB!; isolectotypes: G! [G00364418], K-2!: [K000527990, K000527998], U-2! [U0003349, U0003350], designated by Barneby and Grimes, Mem. New York Bot. Gard. 74(1): 234. 1996.).

#### Pseudalbizzia glabripetala

Taxon classificationPlantaeFabalesFabaceae

(H.S. Irwin) E.J.M. Koenen & Duno comb. nov.

5DB4921F-BAAA-53D4-B4ED-9845484E6CA0

urn:lsid:ipni.org:names:77303819-1

##### Basionym.

*Pithecellobiumglabripetalum* H.S. Irwin, in Mem. New York Bot. Gard. 15(1): 109. 1966.

##### Type.

Guyana. Orealla, Corantyne River, Oct 1879, *G.S. Jenman 364* (holotype: NY! [NY00334664]; isotypes: BM!, P!).

#### Pseudalbizzia inundata

Taxon classificationPlantaeFabalesFabaceae

(Mart.) E.J.M. Koenen & Duno comb. nov.

4C06AB12-BD37-580B-8D43-94C8C87C9632

urn:lsid:ipni.org:names:77303820-1

##### Basionym.

*Acaciainundata* Mart., Spix & Mart. in Reise Bras. 1: 555. 1823.

##### Type.

Brazil. Minas Gerais, Rio Sao Francisco, 1818, *C.F.P. von Martius 1659* (holotype: M! [M0218478]; isotypes: K! [K000797598], NY!).

#### Pseudalbizzia multiflora

Taxon classificationPlantaeFabalesFabaceae

(Kunth) E.J.M. Koenen & Duno comb. nov.

A8A22C6C-E33C-5EFC-A39A-51203902A971

urn:lsid:ipni.org:names:77303821-1

##### Basionym.

*Acaciamultiflora* Kunth, Nov. Gen. Sp. (quarto ed.) 6: 277–278. 1823.

##### Type.

Peru. Cajamarca, Prov. Jaén, San Felipe, 980 m, *Aime Bonpland & F.W.H.A. von Humboldt 3562* (holotype: P! [P00679365]).

**Pseudalbizziamultifloravar.multiflora**

#### Pseudalbizzia multiflora var. sagasteguii

Taxon classificationPlantaeFabalesFabaceae

(Barneby & J.W. Grimes) E.J.M. Koenen & Duno comb. nov.

A92F7886-FF53-5102-A831-F852F3490F16

urn:lsid:ipni.org:names:77303822-1

##### Basionym.

Albiziamultifloravar.sagasteguii Barneby & J.W. Grimes, Mem. New York Bot. Gard. 74(1): 237–238. 1996.

##### Type.

Peru. Cajamarca, Prov. Contumazá, in a quebrada near San Benito, *A. Sagástegui 15410* (holotype: F! [F0042945F]; isotypes: MO! [MO-149743], NY!, US! [US00624358]).

#### Pseudalbizzia pistaciifolia

Taxon classificationPlantaeFabalesFabaceae

(Willd.) E.J.M. Koenen & Duno comb. nov.

469A491C-8055-526E-A64C-B76A0C7E09A0

urn:lsid:ipni.org:names:77303823-1

##### Basionym.

*Mimosapistaciaefolia* [sic] Willd., Sp. Pl. 4: 1028. 1806.

##### Type.

Venezuela. Caracas. *F. Bredemeyer s.n.*, *herb. Willdenow* (holotype: B).

#### Pseudalbizzia subdimidiata

Taxon classificationPlantaeFabalesFabaceae

(Splitg.) E.J.M. Koenen & Duno comb. nov.

22F0902D-975E-5A6A-A042-02DFA0690D44

urn:lsid:ipni.org:names:77303824-1

Albizia subdimidiata (Splitg.) Barneby & J.W. Grimes, Mem. New York Bot. Gard. 74(1): 234. 1996.

##### Basionym.

*Acaciasubdimidiata* Splitg. Tijdschr. Natuurl. Gesch. Physiol. 9: 112 (1842).

##### Type.

Suriname. “ad ripas fluminis Surinami superioris”, 27 April 1838. *Splitgerber 917* (holotype: L [L0018505]).

#### Pseudalbizzia subdimidiata var. minor

Taxon classificationPlantaeFabalesFabaceae

(Barneby & J.W. Grimes) E.J.M. Koenen & Duno comb. nov.

29ACAF5B-D73B-54EC-AEA0-A3DF6CFAED32

urn:lsid:ipni.org:names:77303825-1

##### Basionym.

Albiziasubdimidiatavar.minor Barneby & J.W. Grimes, Mem. New York Bot. Gard. 74(1): 234. 1996.

##### Type.

Guyana. Basin of Essequibo river, Kuyaliwak Falls, 1 Jan 1937, *A.C. Smith 2156* (holotype: NY! [NY00001790]; isotypes: A! [A00069262], G! [G00364427], K! [K000528004], P, U! [U0003358]).

**Pseudalbizziasubdimidiatavar.subdimidiata**

###### Incertae sedis

#### Pseudalbizzia buntingii

Taxon classificationPlantaeFabalesFabaceae

(Barneby & J.W. Grimes) E.J.M. Koenen & Duno comb. nov.

C7F0C7C3-80B8-5C49-9F61-3D3BCD423547

urn:lsid:ipni.org:names:77303826-1

##### Basionym.

*Albiziabuntingii* Barneby & J.W. Grimes, Mem. New York Bot. Gard. 74(1): 223. 1996.

##### Type.

Venezuela. Zulia, alrededores de Casigua El Cubo, 100 m, al este del empalme de la via hacia Casigua con la carretera Machiques-La Fría, 25 Feb 1985, *G.S. Bunting 13370* (holotype: NY! [NY00001782]).

##### Notes.

Fruits of this species are unknown and the species is only known from the type locality (Fig. 5), but it is similar in leaf and inflorescence morphology to several South American species of *Pseudalbizzia*, as described in the protologue. Especially the efoliate pseudoracemes point to this species most likely being correctly accommodated in *Pseudalbizzia*. Collection of fruits and/or inclusion of the species in phylogenetic studies is needed to confirm its generic and sectional placements.

#### Non-native species

Some cultivated and sometimes naturalized Old World *Albizia* species are found in the New World, including: *A.procera* (Roxb.) Benth., *A.julibrissin*, *A.lebbeck* (L.) Benth., and *A.chinensis* (Osbeck) Merr. For these species, Barneby and Grimes (1996) proposed AlbiziasectionAlbizia, now considered as *Albizia* s.s.

## Author contributions

GAP, RR, GCFC, IVM, and RDD designed the study. GAP, LLCI, ELC, RDD contributed labwork. RDD, GCFC, IRM contributed data by supervising students in the lab. EJMK, RDD, XC, SM and CEH contributed taxonomic knowledge, JR contributed species distribution data and the maps. GAP, RDD, ITC, JRP, and RR undertook the phylogenetic analyses. EJMK, RDD, RR, GCFC, CEH and JR contributed to writing the manuscript.

## Supplementary Material

XML Treatment for Pseudalbizzia

XML Treatment for Pseudalbizzia sect. Paniculatae

XML Treatment for Pseudalbizzia barinensis

XML Treatment for Pseudalbizzia coripatensis

XML Treatment for Pseudalbizzia edwallii

XML Treatment for Pseudalbizzia polycephala

XML Treatment for Pseudalbizzia sect. Uninervia

XML Treatment for Pseudalbizzia burkartiana

XML Treatment for Pseudalbizzia sect. Pseudalbizzia

XML Treatment for Pseudalbizzia adinocephala

XML Treatment for Pseudalbizzia berteroana

XML Treatment for Pseudalbizzia sinaloensis

XML Treatment for Pseudalbizzia tomentosa

XML Treatment for Pseudalbizzia tomentosa var. nayaritensis

XML Treatment for Pseudalbizzia tomentosa var. purpusii

XML Treatment for Pseudalbizzia sect. Pterocarpa

XML Treatment for Pseudalbizzia niopoides

XML Treatment for Pseudalbizzia niopoides var. colombiana

XML Treatment for Pseudalbizzia sect. Arthrosamanea

XML Treatment for Pseudalbizzia decandra

XML Treatment for Pseudalbizzia glabripetala

XML Treatment for Pseudalbizzia inundata

XML Treatment for Pseudalbizzia multiflora

XML Treatment for Pseudalbizzia multiflora var. sagasteguii

XML Treatment for Pseudalbizzia pistaciifolia

XML Treatment for Pseudalbizzia subdimidiata

XML Treatment for Pseudalbizzia subdimidiata var. minor

XML Treatment for Pseudalbizzia buntingii

## References

[B1] Alfaro ME Zoller S Lutzoni F (2003) Bayes or bootstrap? A simulation study comparing the performance of Bayesian Markov chain Monte Carlo sampling and bootstrapping in assessing phylogenetic confidence. Molecular Biology and Evolution 20: 255–266. 10.1093/molbev/msg028 12598693

[B2] Baldwin BG Markos S (1998) Phylogenetic Utility of the External Transcribed Spacer (ETS) of 18S–26S rDNA: Congruence of ETS and ITS trees of *Calycadenia* (Compositae). Molecular Phylogenetics and Evolution 10: 449–463. 10.1006/mpev.1998.0545 10051397

[B3] Barneby RC Grimes JW (1996) Silk tree, Guanacaste, Monkey’s earring: A generic system for the synandrous Mimoseae of the Americas. Part I. *Abarema*, *Albizia* and allies. Memoirs of the New York Botanical Garden 74: 1–292.

[B4] Britton NL Killip EP (1936) Mimosaceæ and Caesalpiniaceæ of Colombia. Annals of the New York Academy of Sciences 35: 101–228. 10.1111/j.1749-6632.1933.tb55366.x

[B5] Britton NL Rose JN (1928) North American Flora. Part I. (Rosales). Mimosaceae. 23: 1–194.

[B6] Brown GK Murphy DJ Miller JT Ladiges PY (2008) *Acacia* s.s. and its relationship among tropical legumes, Tribe Ingeae (Leguminosae: Mimosoideae). Systematic Botany 33: 739–751. 10.1600/036364408786500136

[B7] Duno de Stefano R Tun Tun C López Contreras JE Carnevali Fernández-Concha G Leopardi Verde CL Ramírez-Prado JH Can Itza LL Tamayo Cen I (2021) Phylogeny of *Lysiloma* (Fabaceae), a genus restricted to Megamexico with outliers in the West Indies and Florida. Acta Botánica Mexicana 128: e1728. 10.21829/abm128.2021.1782

[B8] Ferm J Korall P Lewis GP Ståhl B (2019) Phylogeny of the Neotropical legume genera *Zygia* and *Marmaroxylon* and close relatives. Taxon 68: 661–672. 10.1002/tax.12117

[B9] Hernández HM (1986) *Zapoteca*: A new genus of Neotropical Mimosoideae. Annals of the Missouri Botanical Garden 73: 755–763. 10.2307/2399204

[B10] Iganci JR Soares MV Guerra E Morim MP (2015) A preliminary molecular phylogeny of the Abarema alliance (Leguminosae) and implications for taxonomic rearrangement. International Journal of Plant Sciences 177: 34–43. 10.1086/684078

[B11] Käss E Wink M (1997) Molecular phylogeny and phylogeography of *Lupinus* (Leguminosae) inferred from nucleotide sequences of the rbc L gene and ITS 1 + 2 regions of rDNA. Plant Systematics and Evolution 208: 139–167. 10.1007/BF00985439

[B12] Katoh K Misawa K Kuma K Miyata T (2002) MAFFT: A novel method for rapid multiple sequence alignment based on Fourier transform. Nucleic Acids Research 30: 3059–3066. 10.1093/nar/gkf436 12136088 PMC135756

[B13] Koenen EJM (2022a) On the taxonomic affinity of *Albiziacarbonaria* Britton (Leguminosae, Caesalpinioideae-mimosoid clade). In: Hughes CE de Queiroz LP Lewis GP (Eds) Advances in Legume Systematics 14. Classification of Caesalpinioideae Part 1: New generic delimitations.PhytoKeys 205: 363–370. 10.3897/phytokeys.205.82288 PMC9848996 36762015

[B14] Koenen EJM (2022b) *Osodendron* gen. nov. (Leguminosae, Caesalpinioideae), a new genus of mimosoid legumes of tropical Africa. In: Hughes CE de Queiroz LP Lewis GP (Eds) Advances in Legume Systematics 14. Classification of Caesalpinioideae Part 1: New generic delimitations.PhytoKeys 205: 453–470. 10.3897/phytokeys.205.82821 PMC9848988 36762017

[B15] Koenen EJM Kidner C de Souza E Simon MF Iganci JR Nicholls JA Brown GK Queiroz LP de Luckow M Lewis GP Pennington T Hughes CE (2020) Hybrid capture of 964 nuclear genes resolves evolutionary relationships in the mimosoid legumes and reveals the polytomous origins of a large pantropical radiation. American Journal of Botany 107(12): 1710–1735. 10.1002/ajb2.1568 33253423 PMC7839790

[B16] Lewis GP Rico Arce L (2005) Tribe Ingeae. In: Lewis G Schrire B Mackinder B Lock M (Eds) Legumes of the World.Royal Botanic Gardens, Kew, Richmond, 193–231.

[B17] Lewis GP Schrire BD (2003) *Thailentadopsis* Kostermans (Leguminosae: Mimosoideae: Ingeae) resurrected. Kew Bulletin 58(2): 491–494. 10.2307/4120634

[B18] Linares L (2005) Especie nueva de *Albizia* (Leguminosae: Mimosoideae) de Centroamérica. Revista Mexicana de Biodiversidad 76: 7–10. 10.22201/ib.20078706e.2005.001.361

[B19] Nielsen IC (1981) Tribe 5. Ingeae. In: Polhill RM Raven PH (Eds) Advances in Legume Systematics.Part 1, Royal Botanic Gardens, Kew, Richmond, 173–190.

[B20] Posada D (2008) jModelTest: Phylogenetic model averaging. Molecular Biology and Evolution 25: 1253–1256. 10.1093/molbev/msn083 18397919

[B21] Rambaut A Drummond AJ Xie D Baele G Suchard MA (2018) Posterior summarization in Bayesian phylogenetics using Tracer 1.7. Systematic Biology 67(5): 901–904. 10.1093/sysbio/syy032 29718447 PMC6101584

[B22] Rico Arce ML Gale SL Maxted N (2008) A taxonomic study of *Albizia* (Leguminosae: Mimosoideae: Ingeae) in Mexico and Central America. Anales del Jardin Botanico de Madrid 65: 255–305. 10.3989/ajbm.2008.v65.i2.294

[B23] Ringelberg JJ Koenen EJM Iganci JR de Queiroz LP Murphy DJ Gaudeul M Bruneau A Luckow M Lewis GP Hughes CE (2022) Phylogenomic analysis of 997 nuclear genes reveals the need for extensive generic re-delimitation in Caesalpinioideae (Leguminosae). In: Hughes CE de Queiroz LP Lewis GP (Eds) Advances in Legume Systematics 14. Classification of Caesalpinioideae Part 1: New generic delimitations.PhytoKeys 205: 3–58. 10.3897/phytokeys.205.85866 PMC9848904 36762007

[B24] Ronquist F Teslenko M van der Mark P Ayres DL Darling A Höhna S Larget B Liu L Suchard MA Huelsenbeck JP (2012) MrBayes 3.2: Efficient Bayesian phylogenetic inference and model choice across a large model space. Systematic Biology 61: 539–542. 10.1093/sysbio/sys029 22357727 PMC3329765

[B25] Soares MVB Guerra E Morim MP Iganci JRV (2021) Reinstatement and recircumscription of *Jupunba* and *Punjuba* (Fabaceae) based on phylogenetic evidence. Botanical Journal of the Linnean Society 196(4): 456–479. 10.1093/botlinnean/boab007

[B26] Soares MVB Koenen EJM Iganci JRV Morim MP (2022) A new generic circumscription of *Hydrochorea* (Leguminosae, Caesalpinioideae, mimosoid clade) with an amphi-Atlantic distribution. In: Hughes CE de Queiroz LP Lewis GP (Eds) Advances in Legume Systematics 14. Classification of Caesalpinioideae Part 1: New generic delimitations.PhytoKeys 205: 401–438. 10.3897/phytokeys.205.82775 PMC9849043 36762006

[B27] Souza ER de Lewis GP Forest F Schnadelbach AS van den Berg C Queiroz LP de (2013) Phylogeny of *Calliandra* (Leguminosae: Mimosoideae) based on nuclear and plastid molecular markers. Taxon 62: 1201–1220. 10.12705/626.2

[B28] Souza ER de Krishnaraj MV Queiroz LP de (2016) *Sanjappa*, a new genus in the tribe Ingeae (Leguminosae: Mimosoideae) from India. Rheedea 26: 1–12.

[B29] Sun Y Skinner DZ Liang GH Hulbert SH (1994) Phylogenetic analysis of *Sorghum* and related taxa using internal transcribed spacers of nuclear ribosomal DNA. Theoretical and Applied Genetics 89: 26–36. 10.1007/BF00226978 24177765

[B30] Terra V Ringelberg JJ Maslin B Koenen EJM Ebinger J Seigler D Hughes CE (2022) Dilemmas in generic delimitation of Senegalia and allies (Caesalpinioideae, mimosoid clade): how to reconcile phylogenomic evidence with morphology and taxonomy? In: Hughes CE de Queiroz LP Lewis GP (Eds) Advances in Legume Systematics 14. Classification of Caesalpinioideae Part 1: New generic delimitations.PhytoKeys 205: 261–278. 10.3897/phytokeys.205.79378 PMC9849036 36762013

[B31] Thiers BM (2016) Index Herbariorum: A global directory of public herbaria and associated staff. New York Botanical Garden’s Virtual Herbarium, New York. http://sweetgum.nybg.org/ih/

[B32] Turland NJ Wiersema JH Barrie FR Greute W Hawksworth DL Herendeen PS Knapp S Kusber WH Li DZ Marhold K May TW McNeill J Monro AM Prado J Price MJ Smith GF [Eds] (2018) International Code of Nomenclature for algae, fungi, and plants (Shenzhen Code) adopted by the Nineteenth International Botanical Congress Shenzhen, China, July 2017. Regnum Vegetabile 159. Koeltz Botanical Books, Glashütten. 10.12705/Code.2018

[B33] Villiers JF (2002) *Viguieranthus*. In: Du Puy D Labat J-N Rabevohitra R Villiers J-F Bosser J Moat J (Eds) The Leguminosae of Madagascar.Royal Botanic Gardens, Kew, Richmond, 271–285.

